# Identification and characterization of a novel *Cytorhabdovirus* associated with goji berry (*Lycium barbarum* L.) crinkle disease

**DOI:** 10.3389/fmicb.2023.1294616

**Published:** 2024-01-04

**Authors:** Rong Wang, Sai Liu, Changqing Xu, Jing Yu, Jianhe Wei, Wanlong Ding, Yong Li

**Affiliations:** Institute of Medicinal Plant Development, Chinese Academy of Medical Sciences and Peking Union Medical College, Beijing, China

**Keywords:** *Lycium barbarum*, virome, next-generation sequencing, novel virus, *Cytorhabdovirus*

## Abstract

Goji berry (*Lycium barbarum* L.) is a traditional Chinese herbal medicinal plant that is extensively cultivated in the arid and semiarid regions of northwest China. In this study, a novel cytorhabdovirus, tentatively named “goji cytorhabdovirus A (GCVA),” was identified from the goji berry plant exhibiting leaf crinkle symptoms through high-throughput sequencing (HTS). GCVA contains a linear, negative sense single-stranded RNA genome of 14,812 nucleotides and encodes six open reading frames in the order of 3′ leader-N-P-P4-M-G-L-5′ trailer. The genome of GCVA shares the highest nucleotide (nt) identity of 65.80% (16% query coverage) with yerba mate virus A (YmVA) (NC_076472). The N and L proteins also share low amino acid (aa) identities (<35.42 and < 41.23%, respectively) with known cytorhabdoviruses. Typical features of the viruses in the genus *Cytorhabdovirus* include a highly conserved consensus sequence in the intergenic regions and extensive complementation of the 5′ non-coding trailer and the 3′ leader. These features were also found in GCVA. These data in combination with a phylogenetic analysis that was based on the aa sequences of the N and L proteins support the proposal that GCVA is a new species in the genus *Cytorhabdovirus*.

## 1 Introduction

Goji (*Lycium barbarum* L.), which is also known as goji berry or wolfberry, is a solanaceous shrub and is extensively cultivated in the arid and semiarid regions of northwest China, including the Ningxia Hui Autonomous Region, Qinghai Province, Gansu Province, Xinjiang Uygur Autonomous Region, and Inner Mongolia (Xin et al., 2013). It is a highly valuable crop. Its fruits (Gou-qi), and root bark (Di-gu-pi) have been used for centuries in traditional Chinese medicine to improve eyesight, liver and kidney function (Wang et al., 2010; Yao et al., 2011). It can also be used as food, and many people avidly use the berries in soups. To our knowledge, almost no viruses have been reported in goji, except for *goji berry chlorosis virus* (GBCV), which was chlorotic identified in another species of goji berry plant (*L. chinense* Miller) (Kwon et al., 2018).

The family *Rhabdoviridae* in the order *Mononegavirales* currently consists of three subfamilies and 46 genera (ICTV, https://ictv.global/taxonomy). Viruses that infect plant hosts and arthropod vectors are grouped in the subfamily of *Betarhabdovirinae*, which are classified into six genera (*Alphanucleorhabdovirus*, *Betanucleorhabdovirus*, *Cytorhabdovirus*, *Dichorhavirus*, *Gammanucleorhabdovirus*, and Var*icosavirus*) based on their replication sites (nucleus or cytoplasm), genome structures (monopartite or bipartite), and vector species (Jackson et al., 2005; Walker et al., 2022). Rhabdoviruses contain a linear, negative sense single-stranded RNA genome that is 11–16 kb long (Dietzgen et al., 2017) and includes five canonical genes in the order of 3′- nucleocapsid protein (N) - phosphoprotein (P) - matrix protein (M) - glycoprotein (G) - polymerase (L) - 5′ (Dietzgen et al., 2020; Walker et al., 2022). The genes are separated by conserved gene junctions, and the entire coding region is flanked by partly complementary 3′ leader and 5′ trailer sequences with transcription and replication initiation signals (Dietzgen et al., 2017). Arthropods, which include aphids (*Aphis fabae* and *A. ruborum*), leafhoppers (*Recilia dorsalis*), planthoppers (*Laodelphax striatellus*) and whiteflies (*Bemisia tabaci*), are common vectors that transmit cytorhabdoviruses in nature (Yang et al., 2017; Cao et al., 2018; Fránová et al., 2019; Dietzgen et al., 2020; Pinheiro-Lima et al., 2020).

**Figure 1:**
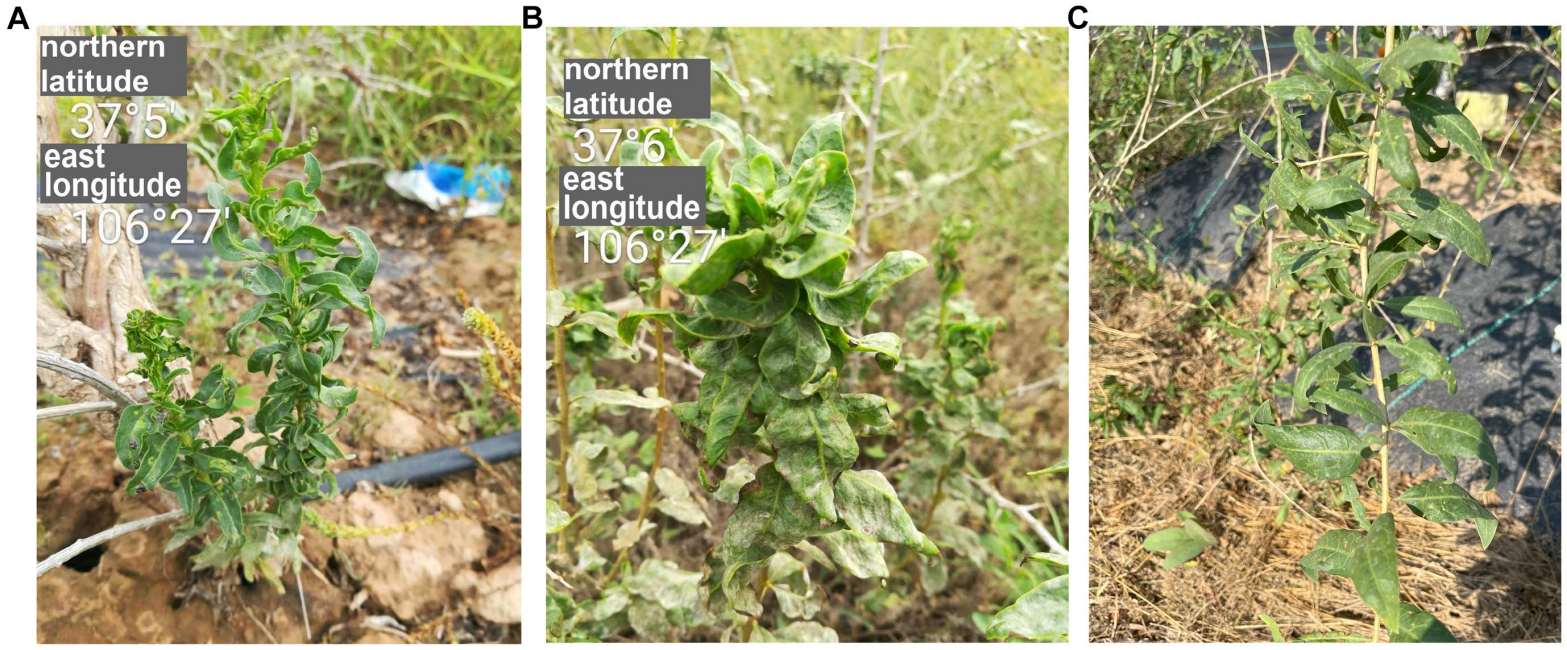
The symptoms of goji berry viral diseases. **(A)** and **(B)** Plants showing symptoms of GCVA infection. **(C)** Healthy plant.

In 2017, goji berry plants with crinkled leaves were found in the Ningxia Autonomous Region, China (Figures 1A,B). Because of their typical symptoms of viral diseases, we analyzed the virus species in the diseased leaves using high-throughput sequencing (HTS). This is a rapidly developing technique that is used to detect and diagnose viral infections, and it identified a novel rhabdovirus that infects plants. The genome of this virus was determined by Sanger sequencing. The virus has a genomic structure that is typical for viruses in the family of *Rhabdoviridae* and is phylogenetically related to cytorhabdoviruses. We proposed to name this virus goji cytorhabdovirus A (GCVA) and classify it as a new member of the genus *Cytorhabdovirus* of the family *Rhabdoviridae*.

## 2 Materials and methods

### 2.1 Plant samples

Goji plants with crinkle leaves (Figure 1A) were found at Tongxin County, Wuzhong City, Ningxia autonomy region, China, in 2017, and were transported to the laboratory for cultivation and preservation. Leaf samples were collected from this plant and were stored at -80°C for paired-end RNA-Seq.

### 2.2 RNA extraction, NGS, and data processing

Total RNA was extracted from the leaf sample from symptomatic goji using a mirVana™ microRNA (miRNA) Isolation kit (Ambion, Thermo Fisher Scientific, Waltham, MA, United States). After treatment with an RNA Clean XP Kit (Beckman Coulter, Brea, CA, United States) and an RNase-Free DNase Set (QIAGEN GmbH, Hilden, Germany), the quality and quantity of total RNA were measured using a NanoDrop spectrophotometer (Thermo Fisher Scientific) and Agilent2100 (Agilent Technologies, Santa Clara, CA, United States). After ribosomal RNA depletion using a Ribo-Zero Magnetic Kit (Epicentre, Lucigen, Middleton, WI, United States), an RNA library was built using a TruSeq RNA Sample Prep Kit (Illumina, San Diego, CA, United States), paired-end sequenced on an Illumina HiSeq X platform according to the manufacturer’s instructions^1^ (Li et al., 2022). Raw sequencing data for the goji library was processed to trim the adaptors and low-quality reads using the FASTX-Toolkit software.^2^ Retained reads were assembled *de novo* using CLC Genomics Workbench 6.0.4 (Qiagen, Valencia, CA, United States) according to the scaffolding contig algorithm. The second assembly was then conducted using CAP3 sequence assembly program.^3^ The final contigs were compared against the NCBI non-redundant (Nr) database using BLASTX with an E-value<1e^−5^ (Li et al., 2022).

### 2.3 Recovery of viral genomes

Viral contigs of 14,839 bp covered most of the genome of the novel virus. The genome of the novel virus was also determined by overlapping reverse transcription (RT)-PCR with primers designed based on the viral contig sequences using Primer Premier 6 (PREMIER Biosoft, Palo Alto, CA, USA) (Supplementary Table S1). The 5′- and 3′-end sequences of viral genomic RNAs were determined by the rapid amplification of cDNA ends-PCR (RACE-PCR) using a SMARTer RACE 5′/3′ Kit (Clontech, Mountain View, CA, USA). For sequencing purposes, PCR amplicons were purified by a TIANgel Midi Purification Kit (TianGen, Beijing, China) and cloned into the pMD18 vector (TaKaRa, Dalian, China), which was used to transform competent *Escherichia coli* DH5α cell. More than five clones per amplicon were sequenced in both directions by the biotechnology company Tsingke (Beijing, China).

### 2.4 Sequence analysis and read assembly

The ORF finder program^4^ was used to analyze viral genome organizations. The Conserved Domain Search Service on the NCBI website^5^ was used to analyze conserved domains of ORFs. The multiple sequence alignment of conserved gene junctions of cytorhabdovirus were performed using CLC Genomics Workbench 21.0.5 (Qiagen). The molecular weight (MW) and isoelectric point (pI) of proteins were computed using Expasy^6^ with default parameters. Signal peptide was predicted using SignalP 6.0 server^7^ The potential transmembrane (TM) topology was predicted using TMHMM Server 2.0.^8^ Pairwise comparisons between viruses were performed using MAFFT program^9^ and displayed by Sequence Demarcation Tool (SDT) software using a color-coded matrix (Muhire et al., 2014).

### 2.5 Phylogenetic analysis

Phylogenetic trees were constructed based on the aa sequences of the L protein and N protein of GCVA, 10 most similar cytorhabdoviruses, two alphanucleorhabdoviruses, two betanucleorhabdoviruses, two dichorhavirus, one gammanucleorhabdovirus, and one varicosavirus. Phylogenetic analyses were performed using the maximum-likelihood method with 1,000 bootstrap replicates in MEGA X (Kumar et al., 2018).

### 2.6 Reverse transcription loop-mediated isothermal amplification (RT-LAMP)

For RT-LAMP, the total RNA of goji leaf was extracted using TRNzol universal reagent (TianGen, Beijing, China) according to the manufacturer’s instructions. The RT-LAMP primers including two outer primers (forward primer F3 and backward primer B3), two inner primers (forward inner primer FIP and backward inner primer BIP), and two loop primers (forward loop primer LF and backward loop primer LB) were designed using the Primer Explorer version 5 software^10^ (Eiken Chemical Co., Tokyo, Japan) (Supplementary Table S1). Before use in LAMP, all primers were assessed for specificity by performing a BLAST search. RT-LAMP was carried out using the Colorimetric pH-sensitive LAMP kit (TransGen Biotech, Beijing, China). The components and their concentrations of RT-LAMP reactions are as follows: FIP/BIP primers 1.6 μM each, F3/B3 primers 0.4 μM each, Loop F/B primers 0.8 μM each, 2.5× pH sensitive LAMP reaction mix 10 μL, N-red stain 1.3 μL, *Bst* II DNA polymerase 1 μL, reverse transcriptase (high temperature) 1 μL, total RNA 1 μL, The volume was refilled to 25 μL with RNase-free ddH_2_O. The isothermal amplification procedure is 62°C for 30 min, 85°C for 10 min.

## 3 Results

### 3.1 Identification of potential viruses infecting the goji berry

In September 2017, goji plants that exhibited severe crinkle symptoms were found in a field (N 37°5′, E106°27′) in Tongxin County, Wuzhong City, Ningxia Autonomous Region, China (Figures 1A,B). No crinkle symptoms were observed on healthy plants (Figure 1C). To identify the viruses in the goji plant, a library was generated using symptomatic leaf sample for paired-end RNA-Seq. A total of 67,439,294 clean reads were obtained. Assembly of the clean reads generated 73,974 contigs that ranged from 200 to 14,839 nucleotides (nt). A BLASTx analysis of the contigs revealed that six contigs (ranged from 565 to 14,839 bp) that obtained from the assembly of 14,076 reads were similar to several viruses in the family of *Rhabdoviridae* (Supplementary Table S2).

### 3.2 Determination of the virus genome

**Figure 2:**
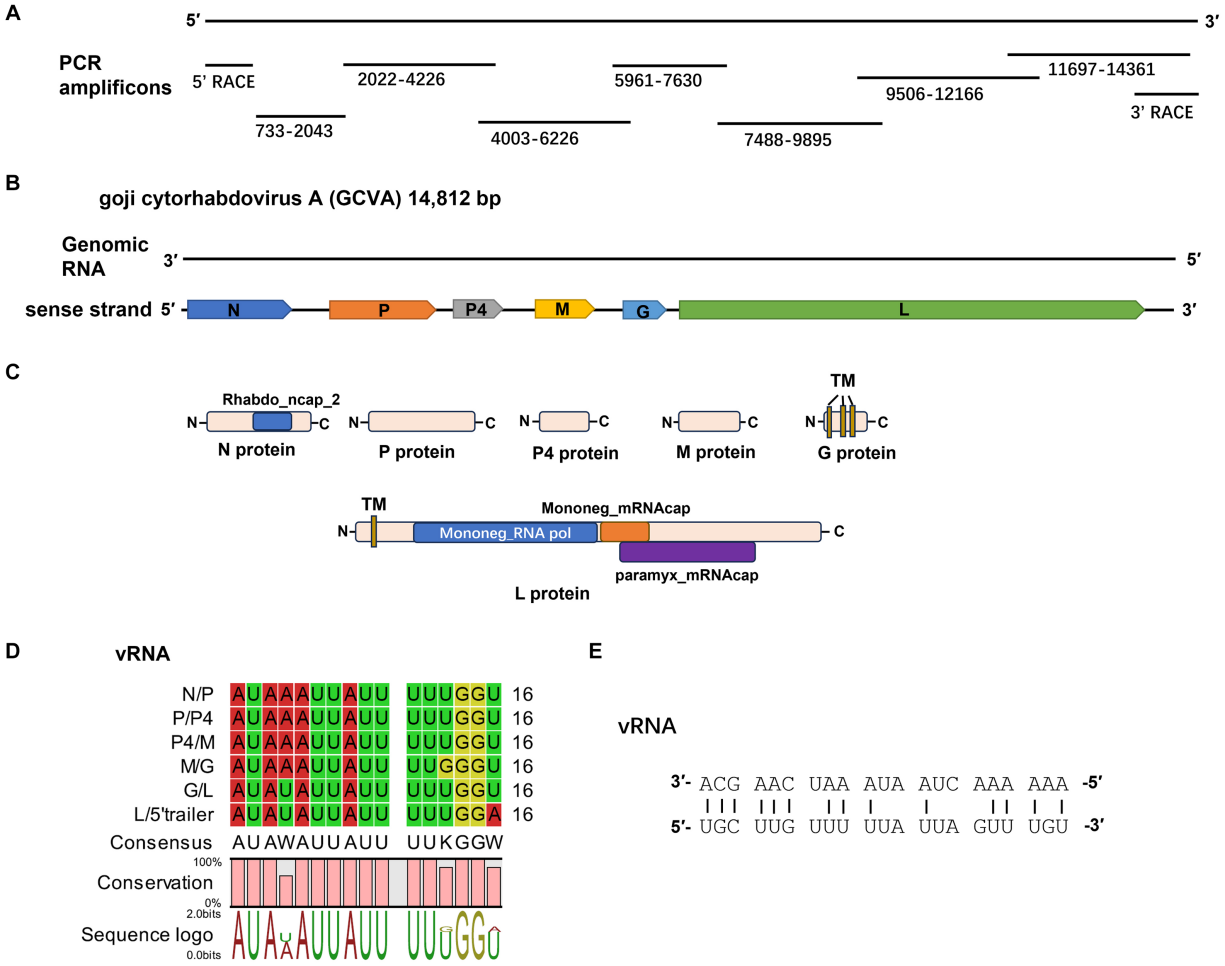
Characterization of a novel cytorhabdovirus, GCVA, isolated from goji berry in China. **(A)** Schematic diagram of genome amplification and sequencing strategy of GCVA. **(B)** Genomic organization of GCVA. N, nucleocapsid protein; P, phosphoprotein; M, matrix protein; G, glycoprotein; L, polymerase. **(C)** Conserved domains of GCVA-encoded proteins. TM, transmembrane topology. **(D)** Conserved gene junction regions of GCVA. **(E)** Complementary terminal nucleotides of GCVA.

To confirm the results of HTS and characterize the rhabdovirus in symptomatic goji plants, the viral genome was determined by amplifying overlapping and terminal cDNA fragments of the new rhabdovirus by reverse transcription PCR (RT-PCR) and rapid amplification of cDNA ends (RACE) PCR with specific primers based on the viral contig sequences (Figure 2A; Supplementary Table S1). The genomic sequence of the novel rhabdovirus was 14,812 nt (GenBank accession number OR489165). A BLASTN analysis with the nucleotide sequence of the genome revealed that this novel rhabdovirus was the most similar to YmVA (NC_076472), which was isolated from yerba mate (*Ilex paraguariensis*) in Argentina with nt identities of 65.80% (16% query coverage). We tentatively named this novel rhabdovirus identified in goji berry as “goji cytorhabdovirus A (GCVA).”

### 3.3 Characterizations of the viral genome

Six open reading frames (ORFs) were identified in the sense strand of GCVA, which has a gene arrangement that is similar to that of most rhabdoviruses (Figure 2B) (Dietzgen et al., 2020).

ORF1 (436–1,950 nt), which follows a short 3′- untranslated region (UTR) of 435 nt, is 1,515 nt long and predicted to encode a 504 amino acid (aa) N protein with a predicted MW of 56.46 kDa and a pI of 7.06 (Table 1). Protein N contains one conserved domain, which was rhabdovirus nucleoprotein (Rhabdo_ncap_2, cl03939) at aa position 222–407 (Figure 2C).

**Table 1:** Features of proteins encoded in the positive-sense orientation by the goji cytorhabdovirus A (GCVA) anti-genome.

ORF	Position (nt)	Protein	Size (nt)	Size (aa)	MW (KDa)	pI^a^	BLASTp match in NCBI^b^	Identity (%)	TM^c^	Signal peptide (cleavage site, Probability)
ORF1	436–1,950	N	1,515	504	56.46	7.06	nucleocapsid (YmVA)	35.42	ND	ND
ORF2	2,499–4,046	P	1,548	515	58.38	7.11	phosphoprotein (YmVA)	26.96	ND	ND
ORF3	4,305–5,024	P4	720	239	26.86	8.34	P4 protein (YmVA)	34.50	ND	ND
ORF4	5,480–6,340	M	861	286	32.61	5.68	–	–	ND	ND
ORF5	6,742–7,368	G	627	208	24.35	8.53	–	–	aa 7–26, 67–89, 102–124	aa 26 and 27, 65.22%
ORF6	7,562–14,344	L	6,783	2,260	259.79	7.00	polymerase (YmVA)	41.23	aa 13–33	ND

a pI, Isoelectric point.

b -, no match.

c TM, transmembrane topology; ND, not detected.

ORF2 (2,499–4,046) is 1,548 nt long and predicted to encode a putative P protein of 515 aa with a predicted MW of 58.38 kDa and a pI of 7.11 (Table 1).

ORF3 (4,305–5,024) is 720 nt long and predicted to encode a putative P4 protein of 239 aa with a predicted MW of 26.86 kDa and a pI of 8.34 (Table 1).

ORF4 (5,480–6,340) is 861 nt long and predicted to encode a putative M protein of 286 aa with a predicted MW of 32.61 kDa and a pI of 5.68 (Table 1).

ORF5 (6,742–7,368) is 627 nt long and predicted to encode a putative G protein of 208 aa with a predicted MW of 24.35 kDa and a pI of 8.53 (Table 1). Three TM helices and topologies were predicted in the G protein at the 7–26, 67–89, 102–124 aa positions, respectively (Figure 2C; Table 1). A signal peptide was also predicted in the putative G protein, which appeared to have a cleavage site between aa 26 and 27 with a probability of 65.22% (Table 1).

ORF6 (7,562–14,344) is 6,783 nt long and predicted to encode an L protein of 2,260 aa. The L protein has three conserved domains, including mononegaviral RNA dependent RNA polymerase (Mononeg_RNA pol, cl15638; aa positions 283–1,173), mononegaviral mRNA-capping region V (Mononeg_mRNAcap, cl16796; aa positions 1,192–1,426), and mRNA capping enzyme (paramyx_mRNAcap, cl44358; aa positions 1,286–1,492) (Figure 2C). One TM topology was predicted in the L protein at the 69–91 aa positions (Figure 2C; Table 1).

The 5′ UTR is 468 nt in size. The gene order 3′ N-P-P4-M-G-L 5′ is consistent with that of plant rhabdoviruses, whose genomes have five conserved canonical genes in the order 3′ N-P-M-G-L 5′ (Dietzgen et al., 2020). P4 is an accessory gene that is located between P and M.

A BLASTp analysis showed that the N, P, P4 and L proteins of GCVA were the most homologous to those of YmVA with 35.42, 26.96, 34.50, and 41.23% aa sequence identities, respectively (Table 1).

The intergenic regions contain the highly conserved consensus sequences “3′ AUAA(U)AUUAUUUUU(G)GGU(A) 5′” (Figure 2D). In addition, the 5′ non-coding trailer sequence had extensive complementarity to the 3′ leader with 14 out of 21 nt (Figure 2E), which is consistent with the characteristics of plant rhabdoviruses (Dietzgen et al., 2017).

**Figure 3:**
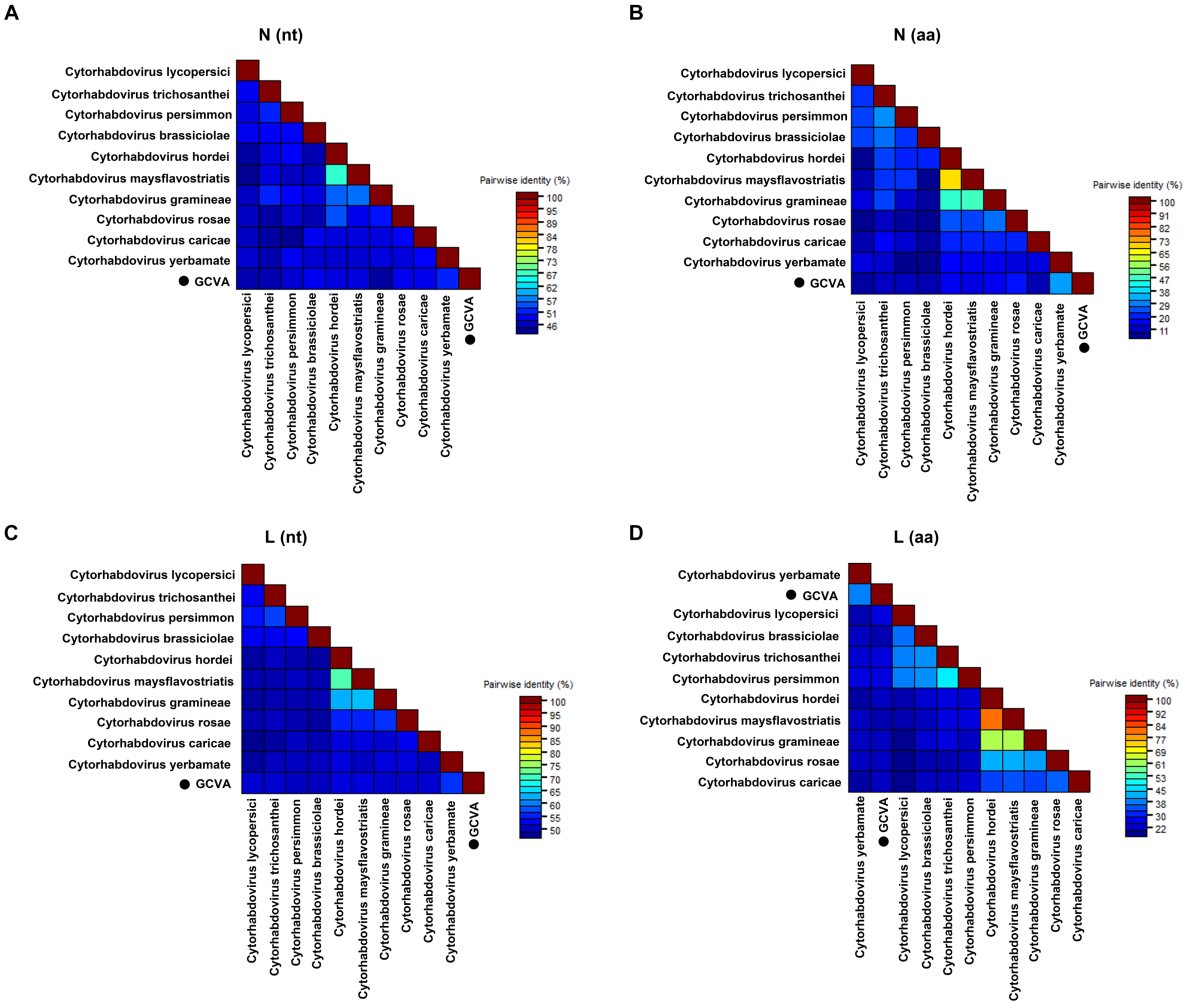
The pairwise identities plot of N and L protein of GCVA and 10 most similar cytorhabdoviruses based on nucleotide (nt) sequence **(A) (C)** and amino acid (aa) sequence **(B) (D)** aligned by MAFFT and displayed by SDT software. N, nucleocapsid protein; L, polymerase.

Pairwise comparisons between GCVA and the 10 most similar cytorhabdoviruses were performed based on the nt and aa sequences of the *N* and *L* genes using the MAFFT program and SDT software, respectively. The *N* gene of GCVA shared 44.5–51.6% nt sequence identities and 9.0–31.7% aa sequence identities with *N* genes in the other aligned cytorhabdoviruses (Figures 3A,B; Supplementary Table S3). The *L* gene of GCVA shared 49.8–55.6% nt sequence identities and 20.2–37.4% aa sequence identities with the *L* genes in the other aligned cytorhabdoviruses (Figures 3C,D; Supplementary Table S3).

### 3.4 Phylogenetic analysis

**Figure 4:**
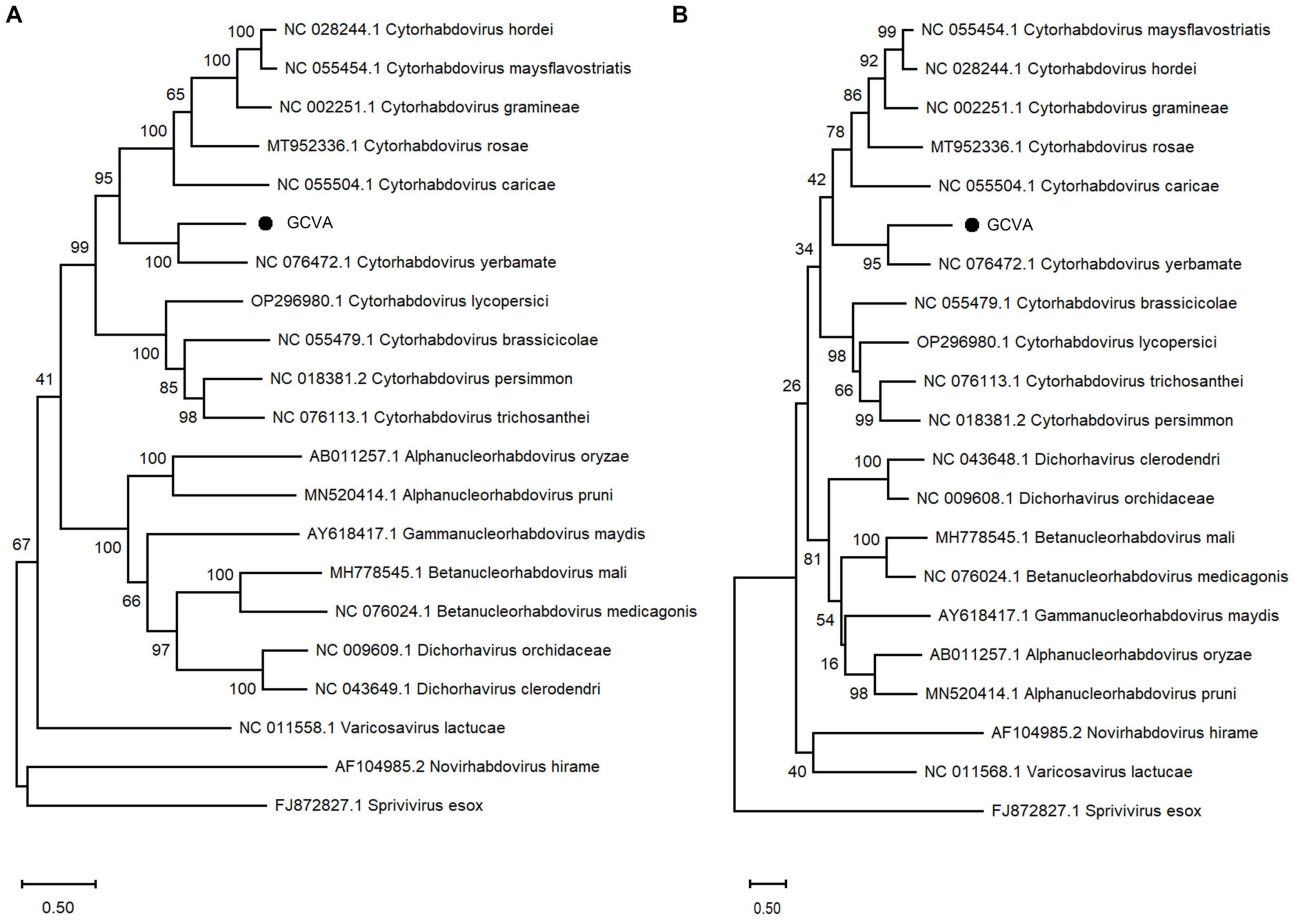
Phylogenetic analysis based on the amino acid sequences of the L **(A)** and N **(B)** of GCVA and 10 most similar cytorhabdoviruses, 2 alphanucleorhabdoviruses, 2 betanucleorhabdovirus, 2 dichorhaviruses, 1 gammanucleorhabdovirus, 1 varicosavirus, 1 novirhabdovirus and 1 sprivivirus, performed by the maximum-likelihood method in MEGA X software. Bootstrap values (1,000 replicates) are shown below the branches. The black triangles represent GCVA identified in this study.

A phylogenetic analysis of the L protein showed that GCVA grouped closely with the other cytorhabdoviruses, particularly *Cytorhabdovirus yerbamate* (Figure 4A). A similar result was obtained in a phylogenetic analysis of the N protein (Figure 4B). These results demonstrate that GCVA should be classified as a new species in the genus *Cytorhabdovirus* in the family of *Rhabdoviridae*.

### 3.5 RT-PCR detection of GCVA in goji berry

To identify the incidence of GCVA in goji berry, 22, 10 and 10 crinkle leaf samples of goji berry were collected from the cultivation area of goji berry in Beijing (BJ), Zhongwei city in Ningxia Hui Autonomous Region (NX), and Golmud city in Qinghai Province (QH), respectively, for RT-PCR detection. The samples were numbered BJ1–BJ22, NN1–NX10, and QH1–QH10, respectively. The specific primer pair (GQ-11380F and GQ-12178R) for GCVA detection and the general primer pair for GCVA and YmVA detection were designed and shown in Supplementary Table S1. The RT-PCR detection results showed that 4 of the 42 samples were positive for GCVA with a detection rate 9.5%, two of which were collected from Beijing, and the other two were collected from Qinghai province (Supplementary Figure S1).

Almost complete genome sequences (755–14,345 nt) of one GCVA BJ isolate (GCVA-BJ) were amplified and sequenced. The sequences have been uploaded to GenBank (GenBank accession number OR860428). The nucleotide sequences alignment revealed that GCVA-BJ isolate shared 98.93 and 45.14% identity with GCVA-NX and YmVA, respectively (Supplementary Figure S2 and Supplementary Table S4).

### 3.6 Development RT-LAMP as a visual diagnostic platform for the detection of GCVA

**Figure 5:**
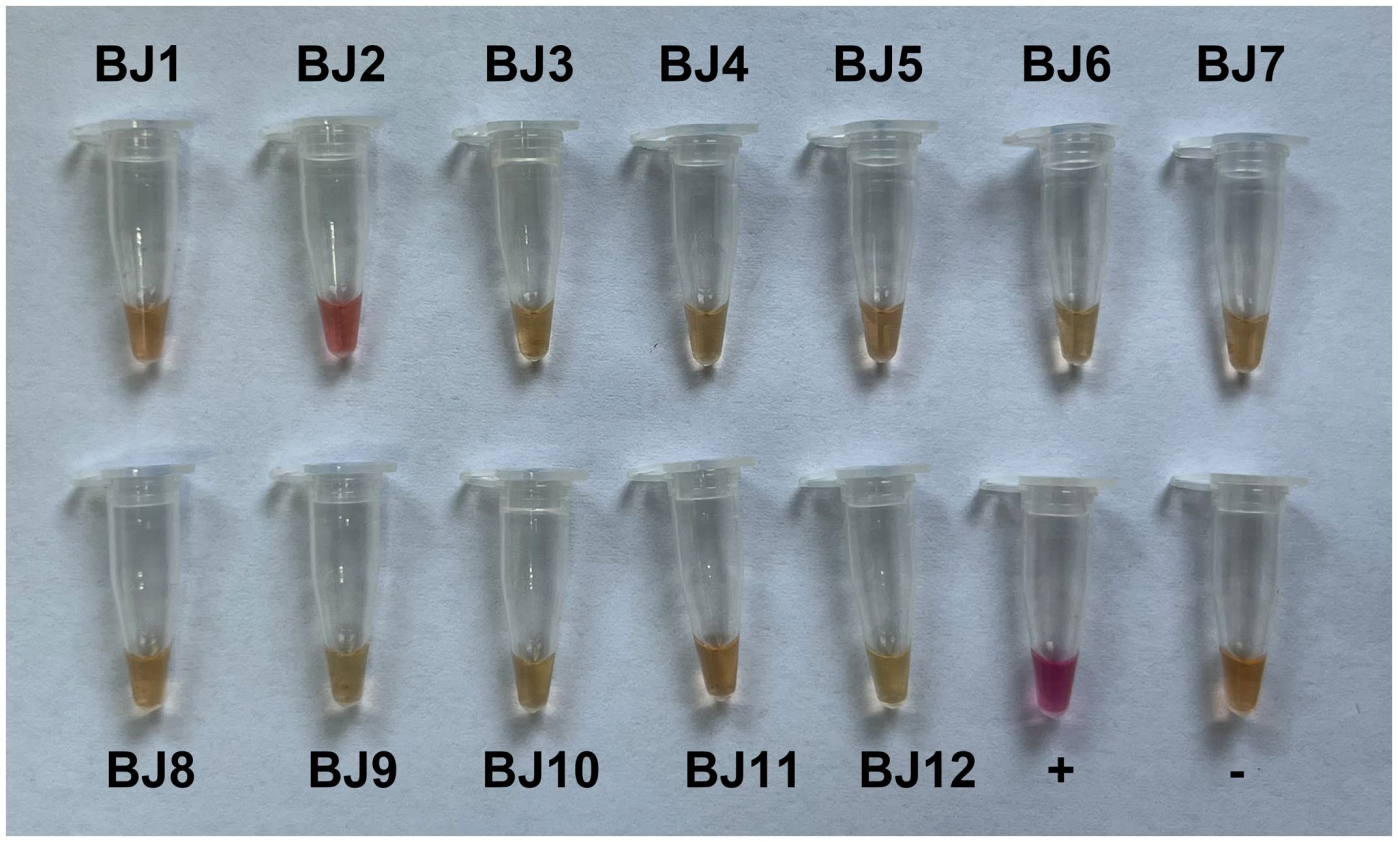
RT-LAMP assay detection of goji cytorhabdovirus A (GCVA) in goji berry.

LAMP is a sensitive and rapid nucleic acid amplification method that is studied widely for the detection of many infectious diseases in the field (Soroka et al., 2021). LAMP allows biosensing of target DNA or RNA under isothermal conditions with high specificity in a short period (Notomi et al., 2000). In this study, we developed a RT-LAMP assay with a set of six primers, F3-176, B3-176, FIP-176, BIP-176, LF-176 and LB-176 (Supplementary Table S1), that specifically amplifies the N gene of GCVA. Since the N-red staining solution was added to the reaction solution, the results could be observed by the naked eye, and the positive samples were magenta, while the negative samples were orange-yellow. Twelve goji berry leaf samples (BJ1–BJ12) were detected using the established RT-LAMP assay, of which only one sample was positive, and the result was consistent with the RT-PCR detection (Figure 5 and Supplementary Figure S1).

## 4 Discussion

Goji berry is perennial solanaceous defoliated shrub, which is cultivated extensively in the arid and semiarid regions of northwest China. To our knowledge, apart from the goji berry chlorosis virus (GBCV), which is classified in an intermediate position between the families *Benyviridae* and *Virgaviridae* (Kwon et al., 2018), there are almost no reports of viral diseases on goji berry. In this study, HTS combined with conventional Sanger sequencing was used to identify a novel virus, GCVA, in a goji berry plant that exhibited typical virus symptoms (Figure 1). The genome of GCVA consists of 14,812 nt and is predicted to encode six ORFs in the order 3′ leader-N-P-P4-M-G-L 5′ trailer (Figure 2B), which conforms to the basic canonical organization (3′ leader-N-P-M-G-L 5′ trailer) described for plant rhabdoviruses (Dietzgen et al., 2020). GCVA shared the highest genomic sequence identity (65.80%) with YmVA (NC_076472), and its N and L proteins were 9.0–31.7% and 20.2–37.4% homologous at the aa level with the 10 most similar cytorhabdoviruses available in the GenBank database (Figures 3B,D; Supplementary Table S3), which met the criteria for species demarcation in the genus *Cytorhabdovirus* (Wang et al., 2021). In the phylogenetic analysis deduced from the aa sequences of proteins L and N, the novel virus clustered together with YmVA within the cytorhabdovirus group (Figures 4A,B). These results show that GCVA should be considered a new species in the genus *Cytorhabdovirus*.

N, P, M, G and L are the five conserved canonical genes in the genomes of plant rhabdoviruses (Walker et al., 2011). One or several accessory genes that may intersperse among or overprint with the canonical genes lead to diverse viral genome organizations containing up to 6–10 genes (Walker et al., 2011). An accessory gene, P4, is located between the P3 and M proteins in the genome of GCVA. A BLASTp analysis showed that P4 of GCVA shares the highest identity with that of YmVA with 34.50% aa sequence identities (Table 1). Unlike the P4 protein of YmVA, TM topology was not predicted on the P4 protein of GCVA (Bejerman et al., 2017).

ORF4 and ORF5 were hypothesized to encode the putative M and G proteins, respectively, based on their locations in the genome. However, the BLASTp analysis did not find any protein that was homologous to the putative M and G proteins (Table 1). Fortunately, three TM topologies were identified in the putative G protein in GCVA at the 7–26, 67–89, 102–124 aa positions (Figure 2C; Table 1), which is similar to the G proteins of the other plant rhabdoviruses and consistent with their membrane functions (Bejerman et al., 2017, 2020). A signal peptide was also predicted in the putative G protein (Table 1), which is similar to the G proteins in several cytorhabdoviruses (Bejerman et al., 2020). These results support the concept that ORF5 may encode a G protein. Since the M protein has no special structural features, we can only hypothesize that the protein encoded by ORF4 is an M protein based on its position in the genome. These may highlight the distinctiveness of GCVA to some extent.

The relative abundance of each viral mRNA is regulated by the transcription termination polyadenylation (TTP) signal at the end of each viral gene. Once the transcriptase reaches this signal, it dissociates from the RNA template (Abraham and Banerjee, 1976). Rhabdoviruses possess conserved TTP signal-like poly(U) tracts in their intergenic regions (Dietzgen et al., 2012). Several highly conserved sequences with a consensus of “3′-AUAA(U)AUUAUUUUU(G)GGU(A)-5′” were also found in the intergenic regions of GCVA RNA (Figure 2D). Although, to the best of our knowledge, this conserved sequence is not similar to those of other rhabdoviruses, they also contain poly(U) tracts that should be TTP signals. The extensive complementarity between the 5′ non-coding trailer and 3′ leader was also found in GCVA, which is consistent with the characteristics of plant rhabdoviruses (Dietzgen et al., 2017).

The occurrence of GCVA in goji berry was preliminarily identified, only 4 of 42 randomly selected leaf samples were positive for GCVA (Supplementary Figure S1), which indicate that GCVA is not popular in goji berry.

Cytorhabdoviruses have been reported to be transmitted by aphids (*Aphis fabae* and *A. ruborum*), leafhoppers (*Recilia dorsalis*), planthoppers (*Laodelphax striatellus*) and whiteflies (*Bemisia tabaci*) in nature (Yang et al., 2017; Cao et al., 2018; Fránová et al., 2019; Dietzgen et al., 2020; Pinheiro-Lima et al., 2020). Psyllid (*Bactericera gobica*), gall mite (*Aceria pallida*), aphids (*Aphis gossypii*), gall midge (*Jaapiella* sp.), and thrips (*Psilothrips indicus*) are five common pests in goji berry (Xu et al., 2014; Li et al., 2017). Further studies are needed to detect whether arthropods harbor GCVA and to determine whether these arthropods can transmit GCVA.

In conclusion, this study is the first to report the complete nucleotide sequences of GCVA that infects goji berry. This novel virus has a genomic organization that is similar to those of members in the genus *Cytorhabdovirus* and shows low levels of genomic homology with reported cytorhabdoviruses. Thus, it should be considered to be a new species in the genus *Cytorhabdovirus*.

## Data availability statement

The datasets presented in this study can be found in online repositories. The names of the repository/repositories and accession number(s) can be found in the article/Supplementary material.

## Author contributions

RW: Formal analysis, Funding acquisition, Investigation, Methodology, Writing – original draft. SL: Resources, Writing – review & editing. CX: Resources, Writing – review & editing. JY: Resources, Writing – review & editing. JW: Project administration, Supervision, Writing – review & editing. WD: Formal Analysis, Investigation, Writing – review & editing. YL: Formal analysis, Methodology, Writing – review & editing.

## Footnotes

^1^www.illumina.com

^2^http://hannonlab.cshl.edu/fastx_toolkit/index.html

^3^http://doua.prabi.fr/software/cap3

^4^https://www.ncbi.nlm.nih.gov/orffinder

^5^www.ncbi.nlm.nih.gov/Structure/cdd/wrpsb.cgi

^6^https://web.expasy.org/protparam/

^7^https://services.healthtech.dtu.dk/services/SignalP-6.0/

^8^https://services.healthtech.dtu.dk/services/TMHMM-2.0/

^9^https://mafft.cbrc.jp/alignment/server/

^10^http://primerexplorer.jp/lampv5e/index.html
