# Supplementary material for: Identification and characterization of a novel Cytorhabdovirus associated with goji berry (Lycium barbarum L.) crinkle disease
Source: Front Microbiol. 2024 Jan 4;14:1294616. doi: 10.3389/fmicb.2023.1294616 (PMC10794335; doi:10.3389/fmicb.2023.1294616)
Supplement: Supplementary file 1 [file Table_1.docx]

>GCVA [organism= Goji cytorhabdovirus A] strai NX, complete sequence

ACGAACAAAAATAATCAAACAAACTTTTATTTAGAGAGAATTCTATCAGAGTTCTTATTGTGAAGTTGTGCACACATTAATGCATGACCCTGGAAGATGATGTCCTTTCAGAGAAAAACGTCCTGTTGTGTGACAGGCAATGCCGATTGTATGCCCGAGTCGGAGTTCGCAGATGACAATGCGGAATAATAGGGGAATTCCCGAGACAACGGGTTTTCGCACCCTAGCGATTCCACCCATTTCGGCGTTCGCCATTCAACTATTACCATCAACATGGAGATCATATTCACTTGAACCAGTGTCAATTATGCCGATTTGATTTACTATATATAATAAAAACCAGGATCATACAATTGGTATTCCTATATATCTATTTTCAACTGTCTCCTTACGAGATTATTTACTGTATAAGGTTAGTTCTATTACGTAATTAAAATGGCCAATACTAGCGGGACCAAGCAAGAGACAATCGAAGAGATACTCTACAAAAAGTATGAGAAGACCCCAGACCCGATCCTCACTAATCTTAGTGAAATCACATACTCAGAGGAAGGAATAAAAAGCAGACGGATTTACCGATTGAGCCCTGACACTGTTTTATCTAATGATCAATTAAAGGCTGCAGGGTCTCTTCTTGTCGAAAAACTTAGAACAGGTACCGATCCTGATCTGGCGAAGTTGGTTTTCTTATTGGCGATGAATATAAGGGATACATCTGACTCCAACCGTTTAATATTCGGAGATCTCAACTGGCCTAACAAAGCAGATATTGATCTGGCTGCCATGAACATTGGATGGAAAGCGTCGCAAACTGTGTCTTCCACTTCTTCGGGGCAGAATAGAGAGCCATTTAAAAGGAAGACAGATGTTGAGCTTGAAATTGAAGCATTAAAGAGCTACGTTGAGCCTGATGTGACTAATTTGACTCCTGAGGTGGCTGAGTCAAAGAAAGCTGCTTCAAAGGCTATTCATCTGAAATCTGCTATAGCAGCAGCTAAGCTTGTGGAGGATAAAAAGGAGGCTGAGTATAATAAATCTACTGCACAGCCAACTGTTTCTAGCCATGATAATGCGTTTGCATCCCCTGAATATTGGCCTTACTTCGCAGCTTACTTGATGAAATGCATGATAAAGACTCCTGCTAATGTCATTGCAGGAAAAGAGAAGCTAAGAGAGCACTTTATAGGGTTTTATCCTGGAAGTGTTGCACAAGCTGTGGATTTTAGTACTGATGTGGTACATCGACTTTCACTCCGATTGAAGGCAGATCAGCCGATAATTGCCACTTGGTTGGGCCACGCATCTGAGTTCGAGGATAATGAGCCTGCTACTACCCAGAACGCTGGGTTGATCAGATACTTGGTCAATATCCAATTCAGTTTTAATGGGATGGCAGGGTATTCTTTATTCAAGGAGGTCATGAACGTTACCAGGTGGAGTGCCAAAGATGCTTTAGTAAAGTTATGGATGGAACCGAATAGAAAAGTGTTGGACACCATAAATGATCTTTTGTCCAATCATGAGAGTGTTATGCTGGAGGGAAAGAAAATTAAGAAAAGCTCAATCTTCAAATACGCGCGAGCAATAGACCCACAGTATTTCTTGTCATTGCAGCCTAGTCAGTGCCTAACCTTAATGTATATCTGCTCCAAGATTCTTACCAACTTCACAGCTTTCAATGATGTAGCTGACCCATTGAATCAAGTTGCATTGAAAAGAATGGGGAAAGGACAAGCAGCTTACCTAGATGTGTTTGTTGATACCGTCCTGCATGATGATGTAGATTCTAATGTTGTCAGAACTAAGATAGAACAGAAAGCTGCACAGAAGTTTCGTGATGTCAAGACAAATGTTGAAGAGAATGAGGAGATGAGTTCTCAGGAATTGTTAATGAAGCAGATGAAGGCAAAGGCAATAGCCTCCTTAAATGCTTCTGATTCAAAGAAGTGATCATTTTCTCTTATCCCTTGAACGAGAGGAATCATCTTATGTAGTGTTTTGTGTTTTTTGATTTTATTCTGAATCTTTGTTTGTTGTATAATCTGCATCATTCTCTAAGACAACTCTCATTGAAATACGACAAATTGATCTATATGTGATTGAAAATTACTACATTGTGATCAATTTAATATTTCAAGTGCATAGAATTTTGGCAAAATATTCCCAATTGGTATCACTAGAATGTTGAATCAGTAACTGACATTCAGGTGATTTATATTGGGAAGTCTATAGTCTTTTCTGCACTTGTAAATATGTTGTATTCTGAGAAAGTTGTCTGAGATAAGGATAGCAGGTTATGTTAGCGTGGTTATTAATTGTGTATGTAAGCTGTCTATTAGCATTAGTACAAGTTTGATCAGTCGTGTGTGTTATTTAATAAAAACCAGGATCTTACAGATATATATTCAATTCCATTAGGTGATAATCTATGTTATTGAAGAGTTCAAATTTTCAAAATTTCTCTTGTATGTTCAACTTTCAGTTAGATGATTCTCTAGATGAACATTCACACTGATATAGTGGGCAAAGGTCTTAAAAACAAGCCGAACTCTTTCTTTAACAACAACTCATTGCCGTTGTTTGTCTTCGATCCTCAAACCACTCCCTCTGATAAATTGCCTATTGTAAAAGCTTATAGCGGACTGTCGAGGAACCAGAGAAGGAAGAACAAAAACTGGGCATTTGATCCAAACCTTACTAGAATAAGAATGGAAAACGCTCAGGCGATGTTTGCAGCTAAATTAGCTGAAGGAGAATTGATAGAAAAAGAATCATATCTCAATTATGTCTTCGATTCTCTGCAGAAAGATGAGGTCCCCTCTTCTCAGACTGATTGGGAGATTCCCAGAAATCTTCCGATAAAGAAATTTTCGTCTCCTGCTACCGCTGATGCAAAGACTGGCACGTCCAGTTGTGAGAATCCTACAAAGAGGTGGGCAGACTCACTTGAAGAAGATCTGGTGGATGAGGCGCTGATCGAGGCAAAAAGAGTAGATGAAGAGCAATTTGATAAAGCCCTAGAATCTGCTGCTAAAGAAGTTGTTGAAGAGGCAATGAAGCCTAATGAGCACACTTTCAGGCCTCCTGGTTTTCGTTCTGCAAAGCCCAAGAAGGCAATGTCACTGCCTGCTTCTAAGCGTGGCCTTAAACCCAGTTATGGTAGGAGGCACGTGAGTCATTCTCCAACTTCTGCAAGATCATCTAGAAGCAGTGAAGAATGTATGGATTACGGTGAGGTCATTTCGATGATAACACACATGTTTAATGCCGAGAATGTGGGTCTAAACAGGGTATTTGAAGATGATTTAGTCCGAATATATAATGAGAAAGGTCATCTTAGTGAGGAGGCTGTCTATTATTATATATGTGGAATAAAAAAAGAAAGACGGAATTCTCTGACCAATAGACAAGAGGCTTTAGTGACACAAATGGCGAATACAACTGCGGCATTTGGGAAGCAGTTGGAAATAATGAAAAGACAAAACGAAGCTTTTGCAGGGAAGATGGATCGTATGAGAGAGCCTACTGAGGTCATCAAGAGGAGAGAAATAGATCTAAAACATTTGAGTAGTGCTCCGACTCAATCTCTATCGAGGACGATCAATCCTGTGCCTATTACACGCGCTCCTCCTGCTAAAGAGACTGCTTACAGACCAAAGGAAAAGGGTATATCTATAAATGAGCCAGTTGTGTCAACACAGCCGAAAGAAATATCCAATGTTAACAAGGAAACAACTGGTGACAGACCGAAACAAGATGCCAAGGATAAAGATCCGGTTGTTCCTGATCCGCTTAGTCACAGACAGCAACTCATGGATGAGCAAGACATAAACAATGTCACAGAGATTGAGAAGATGGAGAACCTGATGTCTGCATTGCAAAATGTTAGCTTTCCCTTCGAATCAATGTCCTCTAAACAGTTACTCCAGTTGATGGAAGTTATTGAGAACAAGTCTCTTGTTAAGATGATGCTCACTGACAATGATGCTGATCGAATGGGCTACATGGGAATGCTGACTGATATTGTGGTTGCAATGAAGATCTAGTCAGGATGTCGGTGAACTAGACCCTGATAATAAAGGCCTTATGGGATTTGTTTTATGGTTTGATTTGTGTTGTTTGAAGTCTTTTTATTATAAGTCATCTGTGAATCATGTTGTAACATGAATAGTAAAGGGTCGTTTTAATGTTGGTGTGTATTTAATAAAAACCAGGATCTATACATGTGTGTGTCACTAGCTTGCTTTAGAATTTAATCAGCTATAATAACTCCTCTTTTGATATTCACTTGAGGCTGATCTGAAATGGACTTGAACATCGATGAGAACAGCACCAAAAGCAAGAAATTGAAGGATGGGAAGATACTATCAGCAGATCTAAACAAAGAGATAATACAAAATATTATCCCGCATAAAGTGCAGTGGTCTTTCTTCTCCTCCTTTCTAAATTCTTCAAACAAAAACCGAACAGTAAGGTGCGATTCTCTGATCGTCTCCTATGCTCCATACATTCAAAATATCTCGGGTTCAATAATCATCAACCTGTATGATTCCAGGCATGAGAATCCAAGGAACAGATTGTTATTGCATACTTCTTTCCCTGCTTCTGAAGACCAGTATATCGCAATACATCCCAATGTTGTTTATAGTGGGGACAACATAGAAGACAGTCTGATGTTGGAAATATTTTCAAAAGATCTTGAGTTGAAAACCTCTAGCACATATGCCTACATAAAGGTAAAATGTGGTTTCTCATCTAGCAGCAAAAGAATGAAAGGTGATGTACCATCATTAGAGGGTGTCCCAGGGTTATATTATACACGTGGAGATCTACTAAATGCTGAAGAGATCTCTAAGCTTACGAGACAACATGGCTTTGTAGGCCCTCCACTTACATCAGGAAATGATTATGTTATCAAAGGAGCAGGAGCAGTCAGCAAGATACAAAAAGAGTTCAAGGCTGATGTGACAGGAAGTAAATATACTCAGAAGAGAACTTATGAAGACAATAACATTGAGCTCTGATCAAGTTGATTTTGTTCACTATATTATAATGGTTCTAATGATGCTACTAGTATTATATATTTGATCTACCTTTGTGGCAAATTTCCTTGATCTTAGTGATGTCTTTCAGTTCAATTAATCATATATCTATTGTATGGCCGATATATTGTTCTTGATGTTTGATGTATTATATAAGGTGGAGTGTCTATCCTGTTTCTGTATTTCGAGTGTTATTTAATAAAAACCAGGATCTCATAGAATTGCCTATCAACATCATCTATAAATCGTTCATTGATGCTGTGGTTATACATTGATTGTAATCACCATATACATAAGCTCTCGTTCACATAAATCATACCACATTGATTGAACAGAGTCGTAGACCTGCACACAAAATAAATATCATAACCTTGATATTAAGGATACCTATATCCATATTGTTAATAACTTACTGTCTCACTGATTACATAAGAATCATGTTGAGATTAGGAGAATCACATAAGGATGTAGATGTGACGGAGTCCCCTGAGAAGAATGTTGACATTGACAATCCTAATTCCAGAAAGATCGGAAAATCAACATCCATCAACATTTCTTCAGATCATCATCCGGTTGACACTAATAAGAAATCCAACTTTGAATTCGTTCGATCTGAAAATCTCAAAATTAATTATTGTGGGATTTTCTCGACTATATCAGGTAGTATAGTAGGTAGAGATAAGGATATGTTGATAATGAATTACAAAGAGATAATAGAAAGATCAATTGATATGGCTCTCAAGTCTGAAAGAGATGTGATCGATGTGAATTGTAAATCTTCCATCTTAACAACTATCATCTGTGAACACATTAGCAATCAGAGAGTGTCTGAAACTATGAGATACATACCATCTCATTTTCTGTTGGGAGATTCAGTATGGGAGTTGAAAATAACCTGTCCATCTACATTGGTGACTCGAGTTCGATATTTACCTAGAGATGAGTCACAATATTCTTTAAAGATCAAGGAAAATGTCATTGAAGATAACAAAGTGAATTACCTATTGAACATAACCGGAGAATTCATAATGTGGAAAGTTCCAGAGGTTCTGGCTTATCAGTTGTATATATCAAATAAAACGACCTTAAGACCTGGTACACTCCTTAATGAACCAACTGATGATGTTACCGTTGTGAGACTTCCTGCTGATGTGAATGAGGAAGAAGAGATAGATTACTCATCTTCATTGACCCTACGTGGGTTGTCAGAAGAGAAGAAAATTCTCAACAATCTGTTCTCCAACAATGACCCTAATCGCTTAGGTAATAACAAGGCTACTTCGAGTAAAATCAAGTGATCACGTATAAGGGTGAAACTGGAATTTATAAGATGAACTTCACTAGAAGATCCATTGTTCTTCTCTTATTTTGCTTATCTTTTATAATAAATAAATAGTATGCTATAATAATATATATCTTGCATATTTTATGTGTGTCGACTTAAGTTAATCGTGTGTGTATCTAAGCTTTCTGGTTGTTATGTTGTCAATCAAAAATAATAATAGTTCTGTTGTGATTTATATAATATGTGTGTACGTGTGTATTTAATAAAACCCACTTAGGATCCTATATCAAGTTAATTTGCTTATAGGTCAGAGTCTATAAAAAGTAATTTGAATTTATCAATTTTAAAGCGTTGAAGCAGATATTATTTTTCATCGAGTCAAATTCTGCACATAACAATGCGAATTATGTATTAATGTTTAACAAGGATCAATTGATAATCTTGTCTTTGAGTACAGTGTACTTGTTATTAATTTTGATCAAGGTAAATGCCCCCTCATGTTTTCTGTTATCAAAATTTGAGAAGGAGTTAGCTCTTGATTCCTTTAGAGAAATGTTTAATGAATTGATTTTTGAGATATCCAAAAAATATAATGAATTGTTGAGAGAGCAGGTGATTGAGTATATGATCTTTATGTTCATGATATTAATGGTGGCAATATTTATATTTCGATTAGGTAATTGCATCATTAAACATATAAATAAAGCAATCATTAAGATAACAATATTGTGTTTATGTTTGGTGTTTAGGATTCCTCAGTCGATTATCAGTGTTGTCGGATTTCTATATAGTGTTTTAAAGCGGATCATGAACATCAATCAATTGGATACTTATCGGATTAGATTATCTAGCACTGGATTGAGCGATTCAGATGTCTCGAATGATGGTTATGATAACACAGATAATAGTTATCAGACATATTTTTCACTCAATAATATGAGAGATATGGTGTTCCTAGATGACAATATGAATGTTTGTGTTCCTGTAACTCACCGAGGAAAAACTTATTATAAGATAACAAAGACACATCATATTGTGTGAACTGCATCATTGTTATCACTGCCAGCGTGCATAGATATGGATAATGATTGGGTGAATAATCTTTATCTGTAGGAATATAACATATATAATAAAAACCAGGAACTTACAGATCTTGTCTTTATAAATATTATTAAACGTTTAATAGTCAGGGTTAAATCATCAACAATTTCTTTAATCACTCATTCTATAAAATATGGATGCGGATTGGTTAGGCTCGATTTTTGGGAACATAGATATGAACAATGATCTTTTCGGAGATCTGATCGATGGGATAGATGAATCCGAAAAAAAGAAATATATGACTGGACTTGGAGACTTTCATCTGAGAAGTGCTATAAAGTGTGTGAACATAGATAGAATTCGTGTCAAAAGGGGGAGATATAGAGTGAATCAAGATTATATCAACTTCATCTCCATATTTCCGAGTGTCACTGTAACTGCTGGAATTCCTGCAGATATATTGGTCTACTTCCTAAGGACCAACGAGTCAATAGTACTTAATAGTCCTGGTGGAGAATATGTCAAGAAAAGAAGTGCAGAGAGGAAACTGACAATTGACATGGTTTGTAGACGTATCACGATGGAGATGAGGAGGGATTCAACTCATCAGGGAGAAACTGGACCGTGCAATTCTCTGAGAAAGAAATTGTTACAGGAATATACCACATTACCAGATTCTACAGGATATTCTTACCTGAGAACTTTGTTTGAGATGTTCGTATTGTGCTCTAGTAGTATAGCATCTAAGAGGCTTCCTCCCGAAGATGCACATAGCCTAGGGGTTCTGAAGGTCAATGAGGAAGGTATTCCGATATATTTAATGATCATGTTAGGGGATCTTTTGATGGTCATTGGCGGCGATCTTTTATTTGTTACCCGAGTGGATCCAGACTACAAAGTAGAAAGGAAATTGGTTTCAGTTACTTCACCTTTTGTGGAGGAAAAGTTGATCTCTTTGTGTGGAGATAACATGAAACACTTAGATAACAGTATATTCGCAAGCCGGTTGGTCAAGGGAGAATGGACATGTTACTCATGTGATGTTCTTCGGATGATTAGTGACAAAATGGCAGAAAGGGATATAGTCTTGCGAAACGCCAGAATTGGGAATTTGATCATAGATTCAGTGTATCCTTCTGAAAGGACAATTAATGATGTTTTTGAAATAGGGGATATACTGATGCTCCATTTAGGAAATGATGCATATAAAGTGATAAAGTGCTATGAGGCTCTGATTACAGGAATTCTCCTCTCTAGATGTGAATCAATCATTTTAGATAAAGAAGAATTCCTTCGAGAGACTATAAGTGATTTGCTCGATGATAATCCAGAGTATGAATGGTCGATTTCAGGATGGTTATCAATAGCAAACAGAATGGCAACAGATCATCATCTATCTCAACTGTATGGATTGTATCGATTATGGGGACATCCGGTTGTAAAATCTACAGATGGATTGCAGAAAGTCAACAGGATTGGCAAGGCAAATAAAACTATCAACAAGTTGATAGCAAACATGGCAGGGATATCTTTTAAAGAGCAACTCTATTCGGGGTATAAGAAGAAGTGGGGGAGATATCCCGCCTTTAAGTTGCTATTGGATAACAGGACTGTTGAGGAATTATATGAAGAATCTTATCTCATCAAATGTCTTGTAGATAATCGATCTTTTGACACAAGAAAGGATGTATACATCCCGTCAGATTGGGATTTAGTTATCAGTCAGAAAACACTGAGTCTACCTGAGACTTTTAATTTGACCATGGTAGTAGATGACAAGGCTATATCTCCTACCAAAGATTACCTCATATCGGTAGCATTGGGTCAAAACCGATTAATGAATCCTTTTGAGAGAAGAGGTGTGTTGAAGTGGATGAACGAGGATTATCATGATTGTCAGGCCTTCTTACAAAAGATCAATGATAACAGTCTGGACTCTAATGACTGTGTGATAGGGCTTTATCCCAAGGAACGAGAATTGAATTCTATACCCAGGATGTTTGCTCTCATGTCTGCTAAGATGAGGAACTATGTTGTTGTGACTGAGCATATGATTGCTGATGATATACTCCCTTTTTTCCCTCAGATAACGATGATGGATGATTTATTGAGTTTGACCAAGAAGATACACGGGGCAACACGCTGCCAGCAAAATAAAGTGAATGCATCAGGGCTTTTTTCAAAGAACAAGTATCTGTTCCAAGTTGACATTTGTTTGAACATGGACTTTGAGAAATGGAACTTAAATATGCGAAAAGAATCCACATATTCAGTTTTCCTAGAGATGGGGAGACTATATGGGATGGATGAGTTGTTCAACAGGACATATGACATTTTTCATGAGAGTTTTGTATATGTTAGCGACGAGAATGCAAAGTTGGAGATTGGATTGGATGACAATGGGGTCCCTCACTTGAGGCCAGACAACGTTCACTCATACACTGGACATATCGGAGGATTTGAAGGCTTGAGACAGAAGGGGTGGACAGTTTTTACTGTAGCGGTGATACAAATGGATTTGAAAGATTTTCCGGTGGCTTACAAATTGATGGGGCAGGGAGATAATCAAGTTCTCATGTTGACTCTAAAGACCAACTCAGTTGACCAATGCGGAAATATAACTGATGATGGAATATTGGAACTAAGAGGACTATTGAAATTGGTGATTAATCGATTGGAATCAGTGTTTTTGGAACTTGGTTTGCCATTAAAGACACTTGAGTCTTGGAGGTCAGAAGAATTTTTCTTATACGGAAAATTCCCTGTGAAGAAAGGTATTCCTCTGAGTATGTCATTGAAGAAACTAAGCAGGTCATTTCCCTTCTCAAATGACGATAGTATGACGATCGACAATGTTATGGGGTCTGTCTTTACAAATGCACAGTCTGCATCCATGTCAGACGTAACCCACTTATTGGCGTATTATTCGGGAATATTTGAGGTCATAAATGGTGCCATGTTAGTTCTAAACTGGCATCCTTTGATCGGACAAGGTTTTTTTGGTTATTTAAAAGAAGGTTTCAACTGGTTCACTTATGAAAATGTTTCATCTGATGACAATAAACGGAGCAAGAAAATATCAATAAGCATACAGGGCACAATGGAGTATTATCTCTTTATTGAATTATTATCTCTAATGCCTAAGTCTCTTGGAGGTTCAAATGGGATAACAGAATATGAATTCTTAATGAGAGGATTTCCTGATAATCAGAGCAGAGACTTGACTTACTTGTGCGAGATCATAAGTAGCAATGTTGAATCAGACAACCCAAAGGAGAGGCAAGTAATATCTGGGCTGATCAATTTTGTCAGATTCAATCTTTCCAATAGCTCTAATCTTGATTTCTTGGTTGAAGATCCCTGTGCTTTAAACTTACTTCAGCCCAAAACACCAATGACCATATTAAGGAAGAAGGTCAAAGACACCTTGACTAAGACAGCCAATTTTAAAAATGAGAATTTCATGGGGTTGTTTAAGCTGTCGATAGATGAGTCCAGAAGATCATTATTAAACAAACTTGCTGAAGGGGATGTATTATTTCCTAGGGTTTTACATGATTGTTATGCAGCTTCCTTGTTTGGGTTTGTTGATGGGATAGTTTCGAAAGTGGATAAGACCGTAACTGTTCAAAGGATCTGTTTGGAGACATCAGATGATGATATAATTAAGGGGCTCTGTCTTGCTGAGAAAAATTATATCAAATATCTTTTCTGGAGATGTGAATTTTATAGAGCAGCTAAGTATGAGAACGAGCCTAAGATCTCCTGTCCCACAAATTATATCAGATGGTTGAGAAACGCTGGATGGAAGAAGGAGGTCCAAGGAGTAACTGTTCCTTATCCTAGTCACACTTTGATGTACAGAGGTAATGATTGTTGCATATCTTGTGATGGAAACGACATCATTACCTGCCATGTATCGGATTTTCTACCAGAGACCACAGAGAGGCTGGTATCATCACTTGGTCAGTCTCCCCCATATCTAGGAAGTTATACAAAAGAGAAAGTGAAGACATATGATAGAGTGGCACTGTACAGCTCTGAGCCGTTGTTAAGAAGAATTGTGCGTATGCTGAGGATCATAGGATGGGGTAATTTAGAGGAATCGAATTTGCATAATTATCTCAGAAATTTACTCAGGTCAGTATGTGATGTAGATGACTCAATATTTCTCTTGAATAAAGAAGATATTGGAGGGTCTCTGGAGCACAGGTATCGAGATAGTGCTTTGAAGCATGGGGCCTTAGCAAGTAACATGTATGGGCTAGGGACATGGATACACATGAGCACGGATAAGTTTGGACAATACACTAAAGGGAGCAAAAATGTCACTTTGCATTTTCAGGCTATATTATGTTGGGTGCAATCAAGAATGTATGAGATCCTCCTGGACTCATCTTTCTCGAATGGAATCGAGTTCAAGGAGTTCCACTTTCATCTTAGTTGTAGTGAATGTATTAAAAGTGTTGAGTATGATGTTCCAGACATCTCTTCAGTCCCTAAAGCTTTGATACCGAAGTTGAAAGATAACCCTTATTGTTATGTTAAGAATGTGATGCTAACTGAAAAAGATAGGTCCGTATATGCAATAAAGGATATCTTTCAGATAGACTCTAATATTGGAATACATAATCTGGATAGCAAAAGTATCAAGTACTTATTTCATGAGTTTTGGGCTGCATCGATATCCAGAGATATCTTTTCTACCTCAGAGACAGATAACACGTCAATAGGAACAGGGGTCCTCGAGATAAATAAATACCCTAGAATTGCATTCTACAGAATTGGAGCTGAACTTTTATATGATTTGATAAGTGACATATCTATCCTATCAGTCATGCGAATGGAAGCGGAGAAGGACACTTACAAAGAATCCGCAATCTCGTTAGACCATTGTGTTAGAGTGTTACTCAATACTTTGAATGAATGTGACAATGAGGGGTTTATCGGGTTGAGTATCCTTTTTTCATGGGAAAATCAAATAAAAGAAATCATGAATTTTACTTCAGTTCTCTTGCCGGGCAGTGTTTCTATGTCTGTAGGTGATTGCTTATTGGCTGCAAAAAGATCATTAATTAATTATGTGGAAAATAAGACAAATTGGTTCAACCACAGATGCAGATATATCAACTTGAATGAGATGACCCCTGTGGATACATCTATACTCTTGAGGTACGCTGCAGAATTTGATTTCGAATCAGTAGAATCTTGTTCTGAGTGTCGAAGGAGTATCTTGCTTAATATATCAAATGAAAAGCTGTTGTCTTTATCAGGAACAGAGAAATGTGAGTATGGTCATATTTGGTTTCATAAGGAGTATACCACTAAATATGTGACTGTGATTTGCATTCCAGAAGATGCATTGTCTAAACAACAATCATTCAGTTATCTTATAAAGACAAAGGTCGTCAAGCAGTCAATCAGAAATAAAGAGTTAAGAAACCATTGTAATACTATGAGACGGTGTTTTTCTCTGAAACCTGTGTTCTCATCAAATGATATTAGATACACTAATGATCCCTCGATAATAGAAGGCAAGATCTTGTTCCAGGGGAATGTGGATCCCATGCGATTGTGCTATAGATGCATACCTACTGAATTGTCATCGGGATATAGATTATTGGACATATTGGTCGGTTTACGTATTCCCGTCATGGAAAGATCCTGCAGAGTCCTTAATTTAGGGGATGGATTTGGCGGAACAGGGTATTTGCTGGAGAAGCTGATAAGATGTCATGTGATAAATGCAACGTTAGTTGATTGCACTAGTGCTTTTCCACAGACATTTCCTAACTCTAGACCATCTAGTCAATACCTGAGTAGTAATTTGTCTCGGTTCGATAATTCCTTAAGCAAGATAATGGTTAATAATATATTTTCTGAAGCGGTTGTCTTAAGGTACAGGCAGGTATGCGAGTTAAGTGGTACAAAATTGGGCATTTGTGATATTGAACTGGAACACAATGAGACTTCAAGCTTCAACAATGTTGGCCGATTCCCATATTCAAGACTGATAAAGCAGTTAAGGAAGATCAATTTAGACATATTCTTAGTTAAGATCCGGGTCAAAAGCAATGTTGAACTTTATCATGTCTTAGAAATTGCAAATTATAACTATCATCATTTTGAGGTCTATATCACCCCGACTTGCAACAACATGAAGGGAGAATTCTTCTTAAGGTTGGAGGGAGCAAAAGAAGAGTTGCCTATAGAAAGTCGGATAATGAATTTCTCCACTAGAAATAAGATATTAAGGCGTTTTATCAATTATACTGGAGGGAAGTCTTTCTCTAATGTTGATAGTGAATACTATTTTGCTCAGAATCTTATCATGATAGACCCGACATTGCAACATAGAATGCTATCTTATGTAAAAGATTGGATAAAGGAACCGAGATTGAACATAGATATGAGCATGAAAAGTTTCACATCTTTGTTCTTATCGATTGCCGCTGCAAGAAATCCCCGATTGTTTGTCCATCACACAGATAAGAAAGACAAGTATGAGTATAATGATGATCTTTTGAGTATTGGATGGAGGATCTTTACTCTCGGGCTTGCAAAAATACAATCTGAGTCAACATGTTGTTCTGCACTTGATAACATTGAGAGTTACCTGCTAATCAGACCTGCTGTGGTTTCAACTGTAAATAAGGTAAAGGTGATAAGAAAATACACTTATATGATAGTCTCTGAGACGGAGTATGAGGATTGCAAATCTAGGAATAACAGGTTATTTGATTATACTACGCTCCCTCCTTTAAGACTGATGAGGAATGAAAGTATCCAACGGATGTTTCCCATCATAAGACTGATAAACATGAGAGATCATATGTTGCAGCAGATAGATAGACTAGATTTGATCGAATTCTCTTATTTATCTAGAAAAAGGAGAGCAGAGAGAGCTTTAGGGGCTCAGAGATCAGTAGTACTGGAGATTTCCAAAACCAGCTGGTTGTCCTTGATAGAAAAATAATATTTGCAATATAGCGTTTAGTGACCTAATTCAAATAGCAATTGGTTTTTATTATATATTATCTATAAGTGTGTTTTTACACGTAAAATGTGGTTATATAATAAAAACCTTGGTTTTTATTATATAATTTCATTGGGTAAGTATATCATGAAATTCATTTCACCTTTATATGATCATATAGATTATGATTGTCTATTTATTCAATGAGAATCAACTAGGTATCTGCGCTTTGTAGAAACGCTAGGGGCCGCTTTCTCCTTAAAAGAGGATCTTGAAGCGCAATTTATCACATTGTCAGTGTGACGGTCACGTATCTTTCGACCTTATGCTCACAATCGAGACTTTCTACTCCGATGGACTTCTCACCGAATGGACCAACATTTACGTGTGAAGGTCAACACGATCGAACAGTCGCCTATTTCAAGATGACTGACGTCTATAGGGGGGTTTTTTGATTATTTAGTTCGT

>GCVA-BJ [organism= Goji cytorhabdovirus A] isolate BJ, partial cds

AATCGAAGAGATACTCTACAAAAAGTATGAGAAGACCCCAGACCCGATCCTCACTAATCTTAGTGAAATCACATACTCAGAGGAAGGGATAAAAAGCAGACGGATTTACCGATTGAGCCCTGACACTGTTTTATCTAATGATCAATTAAAGGCTGCAGGGTCTCTTCTTGTAGAAAAACTTAGAACAGGTACTGATCCTGATCTGGCGAAGTGGGTTTTCTTATTGGCGATGAATATAAGGGATTCATCTGACTCCAACCGTTTAATATTCGGAGATCTCAACTGGCCTAACAAAGCAGATATTGATCTGGCTGCCATGAACATTGGATGGAAAGCGTCGCAAACTGTGTCTTCCACTTCTTCAGGGCAGAATAGAGAGCCATTTAAAAGGAAGACAGATGTTGAGCTTGAAATTGAAGCATTAAAGAGCTACGTTGAGCCTGATGTGACTAATTTGACTCCTGAGGTGGCCGAGTCGAAGAAAGCTGCTTCAAAGGCTATTCATCTGAAATCTGCTATAGCAGCAGCTAAGCTTGTGGAGGATAAAAAGGAGGCTGAGTATAATAAATCTACTGCACAGCCAACTGTTTCTAGCCATGATAATGCGTTTGCATCCCCTGAATATTGGCCTTACTTCGCAGCTTACTTGATGAAATGCATGATAAAAACTCCTGCTAATGTCATTGCAGGAAAAGAGAAGCTGAGAGAGCACTTCATAGGGTTTTATCCTGGAAGTGTTGCACAAGCTGTGGATTTTAGTACTGATGTGGTACATCGACTTTCACTCCGATTGAAGGCAGATCAGCCGATAATTGCCACTTGGTTGGGCCACGCATCTGAGTTCGAGGATAATGACCCTGCTACTACCCAGAACGCTGGGTTGATCAGATACTTGGTCAATATCCAATTCAGTTTTAATGGGATGGCAGGGTATTCTTTATTCAAGGAGGTCATGAACGTTACCAGGTGGAGTGCCGAAGATGCTTTAGTGAAGTTATGGATGGACCCGAATAGAAAAGTGTTGGACACCATAAATGATCTTTTGTCCAATCATGAGAGTGTTATGCTGGAGGGAAAGAAAATTAAGAAAAGCTCAATCTTCAAATACGCGCGAGCAATAGACCCACAGTATTTCTTGTCATTGCAGCCTAGTCAGTGCCTAACCTTAATGTATATCTGCTCCAAGATTCTCACCAACTTCACAGCTTTCAATGATGTAGCTGACCCATTGAATCAAGTTGCATTGAAAAGAATGGGGAAAGGACAAGCAGCTTACCTAGATGTGTTTGTTGATACCGTCCTGCATGATGATGCAGATTCTAATGTTGTCAGAACTAAGATAGAACAGAAAGCTGCACAGAAGTTTCGTGATGTCAGGACAAATGTTGAAGAGAATGAGGAGATGAGTTCTCAGGAATTGTTAATGAATCAGATGAAGGCAAAGGCAATAGCCTCCTTAAATGCTTCTGATTCAAAGAAGTGATCATTTTCTCTTATCCCTTGAACGAGAGGAATCATCTTATGTAGTGTTTTGTGTTTTTTGATTTTACTCTGAATCTTTGTTTGTTTTATAATCGGCATCATTCTCTAAGACAACTCTCATTGAAATACGACGAATTGATCTATATGTGATTGAAAATTACTACATTGTGATCAATTTAATATTTCAAGTGCATAGAATTTTAGCAAAATATTCCCAATTGGTATCACTAGAATGTCGAATTAGTAACTGACATTCAGGTGATTTATATTGGGAAGTCTATAGTCTTTTCTGCACTTGTAAATATGTTGTATTGTGAGAAAGTTTTCTAAGATAAGGATAGCAGGTTATGTTAGCGTGGTTATTAATTGTGTATGTAAGCTGTCTATTAGCATTAGTACAAGTTTGATCAGTCATGTGTGTTATTTAATAAAAACCAGGATCTTACAGATATATATTCAATTCCATTAGGTGATAATCTATGTTATTGAAGAGTTCAAATTTTCAAAATTTCTCATGTATGTTCAACTTTCAGTTAGATGATTCTCTAGATGAACATTCACACTGATATAGTAGGCAAAGGTCTTAAAAACAAGCCGAACTCTTTCTTTAACAACAACTCATTGCCGTTGTTTGTCTTCGATCCTCAAACCACTCCCTCTGATAAATTGCCTATTGTAAAAGCTTATAGCGGTCTGTCGAGGAACCAGAGAAGGAAGAACAAAAACTGGGCATTTGATCCAAACCTTACTAGAATAAGAATGGAAAACGCTCAGGCGATGTTTGCAGCTAAATTAGCTGAAGGAGAATTGATAGAAAAAGAATCATATCTCAATTATGAATTTGATTCTCTGCAGAAAGATGAGGTCCCCTCTTCTCAGACTGATTGGGAGATTCCCAGAAATCTTCCGATAAAGAAATTTTCGTCTCCTGCTACCGCTGATGCAAAGATTGGCACGTCCAGTGGTGAGAATCCTACAAAGAGGTGGGCAGACTCACTTGAAGAAGATCTGGTGGATGAGGCGCTGATCGAGGCAAAAAGAGTAGATGAAGAGCAATTTGATAAAGCCCTAGAATCTGCTGCTAAAGAAGTTGTTGAAGAGGCAATGAAGCCTAATGAGCACACTTACAGGCCTCCTGGTTTTCGTTCTGCAAAGCCCAAGAAGGCAATGTCACTGCCTGCTTCTAAGCGTGGCCTTAAACCCAGTTATGGTAGGAGGCACGTGAGTCATTCTCCAACTTCTGCAAGATCATCTAGAAGCAGTGAAGAATGTATGGATTACGGTGGGGTCATTTCGATGATAACACACATGTTTAATGCCGAGAATGTTGGTCTAAACAGGGTATTTGAAGATGATTTAGTCCGAATATATAATGAGAAAGGTCATCTTAGTGAGGAGGCTGTCTACTATTATATATGTGGAATAAAAAAAGAAAGACAGAATTCTTTGACCAATAGACAAGAGGCTTTAGTGACACAAATGGCGAATACAACTGCGGCATTTGGGAAGCAGTTGGAAATAATGAAAAGACAAAACGAAGCTTTTGCAGGGAAGATGGATCGTATGAGAGAGCCTACTGAGGTCATTAGGAGACGAGAAATAGATCTAAAACATTTGAGTAGTGCTCCGACTCAATCTCTATCGAGGACGATCAATCCTGTGCCTATTACACGGGCTCCTCCTGCTAAAGAGACTGTTTACAGACCAAAGGAAAAGGGTATATCTATAAATGAGCCAGTTGTGTCAACACAGCCGAAAGAAATGTCCAATGTTAACAAGGAAACAACTGGTGACAGACCGAAACAAGATACCAAGGATAAAGATCCGGTTGTTCCTGATCCGCTTAGTCACAGACAGCAACTCATGGATGAGCAAGACATAAACAATGTCACAGAGATTGAGAAGATGGAGAACCTGATGTCTGCATTGCAAAATGTTAGCTTTCCCTTCGAATCAATGTCCTCTAAACAGTTACTCCAGTTGATGGAAGTTATTGAGAACAAGTCTCTTGTTAAGATGATGCTCACTGACAATGATGCTGATCGAATGGGCTACATGGGAATGCTGACTGATATTGTGGTTGCAATGAAGATCTAGTCAGGATATCGGTGAACTAGACCCTGATAATAAAGGCCTTATGGGATTTGTTTTATGTTTTGATTTGTGTTGTTTGAAGTCTTTTTATTATAAGTCATCTGTGAATCATGTTGTAACATGAATAGTAAAGGGTCGTTTTAATGTTGGTGTGTATTTAATAAAAACCAGGATCTATGCATGTGTGTGTCACTAGCTTGCTTTAGAATTTAATCAGCTATAATAACTCCTCTTTTGATATTCACTTGAGGCTGATCTGAAATGGACTTGAACATCGATGAGAACAGTACCAAAAGCAAGAAATTGAAGGATGGGAAGATACTATCAGCAGATCTAAACAAAGAGATAATACAAAATATTATCCCGCATAAAGTGCAGTGGTCTTTCTTCTCCTCCTTTCTAAATTCTTCAAACAAAAACCGAACAGTAAGGTGCGATTCTCTGATCGTCTCCTATGCTCCATACATTCAAAATATCTCGGGTTCAATAATCATCAACCTGTATGATTCCAGCCATGAGAATCCAAGGAACAGATTGTTATTGCATACTTCTTTCCCTGCTTCTGAAGACCAGTATATCGCAATACATCCCAATGTTGTTTATAGTGGGGACAAGATAGAAGACAGTCTGATGTTGGAAATATTTTCAAAAGATCTTGAGTTGAAAACCTCTAGCACATATGCTTACATAAAGGTAAAATGTGGTTTCTCATCTAGCAGCAAAAGAATGAAAGGTGATATACCGTCATTAGAGGGTGTCCCAGGGTTATATTATACACGTGGAGATCTACTAAATGCTGAAGAGATCTCTAAGCTTACGAGACAACATGGCTTTGTAGGCCCTCCACTTACATCAGGAAATGATTATGTTATCAAAGGAGCAGGAGCAGTCAGCAAGATACAAAAAGAGTTCAAGGCTGATGTGACAGGAAGTAAATACACTCAGAAGAGAACTTATGAAGACAAGAACAGTGAGCTCTGATCAAGTTTATTTTATTCACTATATTATAATGGTTCTAATGATGTTACTAGTATTATATATTTGATCTACCATTGTGGCAAATTTCCTTGATCTTAGTGATGTCTTTCAGTTCAATTAATCATATATCTATTGTATGGTCGATATATTGTTCTTGATGTTTGATGTATTATATAAGGTGGAGTGTCTATCCTGTTTCTGTATTTCGAGTGTTATTTAATAAAAACCAGGATCTCATAGAATTGCCTATCAACATAATCTATAAATCGTTCATTGATGCTGTGGTTATACATTGATTGTAATCACCATATACATAAGCTATCGTTCACATAAATCATACCACATTGATTGAACAGAGTCGTAGACCTGCACACAAAATAAATATCATAACCTTGATATTAAGGATACCTATATCCATATTGTTAATAACTTACTGTCTCACTGATTACATAAGAATCATGTTGAGATTAGGAGAATCACATAAGGATGTAGATGTGACGGAGTCCCCTGAGAAGAATGTTGACATTGATAATCCTAATTCCAGAAAAATCGGAAAATCAACATCCATCAACATTTCTTCAGATCATCATCCGGTTGACACGAATAAGAAATCCAACTTTGAATTCGTTCGATCTGAAAATCTCAAAATTAATTACTGTGGGATTTTCTCGACTATATCAGGTAGTATAGTAGGTAGAGATAAGGATATGTTGATAATGAATTACAAAGAGATAATAGAAAGATCAATTGATATGGCTCTCAAGTCTGAAAGAGATGTGATCGATGTGAATTGTAAATCTTCCATCTTAACAACTATCATCTGTGAACACATTAGCAATCAGAGAGTGTCTGAAACTATGAGATACATACCATCTCATTTTCTGTTGGGAGATTCAGTATGGGAGTTGAAAATAACCTGTCCATCGACATTGGTGACCCGAGTTCGATATTTACCTAGAGATGAGTCACAATATTCTTTAAAGATCAAGGAAAATGTCATTGAAGATAACAAAGTGAATTACCTATTGAACATAACCGGAGAATTCATAATGTGGAAAGTTCCAGAGGTTCTGGCTTATCAGTTGTATATATCAAATAAAACGACCTTAAGACCTGGTACACTCCTTAATGAGCCAACTGATGATGTTACCGTTGTGAGACTTCCTGCTGATGTGAATGAGGAAGAAGAGATAGATTACTCATCTTCATTGACCCTACGTGGGTTGTCAGAAGAGAAGAAAATTCTCAACAATCTGTTCTCCAACAATGACCCTAATCGTTTAGGTAATAACAAGGCTACTTCGAGTAAAATCAAGTGATCACGTATAAGGGTGAAACTGGAATTTATAAGATGAACCTCACTAGAAGATCCATTGTTCTTCCCTCATTTTGCTTATCTTTTATAATAAATAAATAGTATGCTGTAATAATATATATCTTGCATATTTTATGTGTGTCGACTTAATTTAATCGTGTGTGTATCTAAGCTTTCTGGTTGTTATGTTGTCAATCAAAAATAATAATAATTCTGTTGTGATTTATATAATATGTGTGTACGTGTGTATTTAATAAAACCCACTTAGGATCCTATATCAAGTTAATTTGCTTATAGGTCAGAGTCTATAAAAAGTAATTTGAATTTATCAATTTTAAAGCGTTGAAGCAGATATTATTTTTCATCGAGTCAAATTCTGCACATAACAATGCGAATTATGTATTAATGTTTAACAAGGATCAATTGATAATCTTGTCTTTGAGTACAGTGTACTTGTTATTAATTTTGATCAAGGTAAATGCCCCCTCATGTTTTCTGTTATCAAAATTTGAGAAGGAGTTAGCTCTTGATTCCTTTAGAGAAATGTTTAATGAATTGATTTTTGAGATATCCAAAAAATATAATGATTTGTCGAGAGAGCAGGTGATTGAGTATATTATCTTTATGTTCATGATATTAATGGTGGCAATATTTATATTTCGATTAGGTAATTACATCATTAAACATATAAATAAAGCAATCATTAAGATAACAATATTGTGTTTATGTTTGGTGTTTAGGATTCCTCAGTCGATTATCAGTGTTGTCGGATTTCTATATAGTGTTTTAAAGCGGATCATGAACATCAATCAATTGGATACTTATCGGATTAGATTATCTAGCACTGGATTGAGCGATTCAGATGTCTTGAATGATGGTTATGATAACGCAGATGATAGTTATCAGACATATTTTTCACTCAATAATATGAGAGATATGGTGTTCCTAGATGACAATATGAATGTTTGTGTTCCTGTAACTCACCGAGGAAAAACTTATTATAAGATAACAAAGACACATCATATTGTGTGAACTGCATCATTGTTATCACTGCCAGCGTGCATAGATATGGATAATGATTGGGTGAATAATCTTTATCTGTAGGAATATAACATATATAATAAAAACCAGGAACTTACAGATCTTGTCTTTATAAATATTATTAAACGTTTAATAGTCAGGGTTAAATCATCAACAATTTCTTTAATCACTCATTCTATAAAATATGGATGCGGATTGGTTAGGCTCGATTTTTGGGAACATAGATATGAACAATGATCTTTTCGGAGATCTGATCGATGGGATAGATGAATCCGAAAAAAAGAAATATATGACTGGACTTGGAGACTTTCATCTGAGAAGTGCTATAAAGTGTGTGAACATAGATAGAATTCGTGTCAAAAGGGGGAGATATAGAGTGAATCAAGATTATATCAACTTCATCTCCATATTTCCGAGTGTCACTGTAACTGCTGGAATTCCTGCAGATATATTGGTCTACTTCCTAAGGACCAACGAGTCAATAGTACTTAATAGTCCTGGTGGAGAATATGTCAAGAAAAGAAGTGCAGAGAGGAAACTGACAATTGACATGGTTTGTAGACGTATCACGATGGAGATGAGGAGGGATTCAACTCATCAGGGAGAAACTGGACCGTGCAATTCTCTGAGAAAGAAATTGTTACAGGAATATACCACATTACCAGATTCTACAGGATATTCTTACCTGAGAACTTTGTTTGAGATGTTCGTATTGTGCTCTAGTAGTATAGCATCTAAGAGGCTTCCTCCCGAAGATGCACATAGCCTAGGGGTTCTGAAGGTCAATGAGGAAGGTATTCCGATATATTTAATGATCATGTTAGGGGATCTTTTGATGGTCATTGGCGGCGATCTTTTATTTGTTACCCGAGTGGATCCAGACTACAAAGTAGAAAGGAAATTGGTTTCAGTTACTTCACCTTTTGTGGAGGAAAAGTTGATCTCTTTGTGTGGAGATAACATGAAACACTTAGATAACAGTATATTCGCAAGCCGGTTGGTCAAGGGAGAATGGACATGTTACTCATGTGATGTTCTTCGGATGATTAGTGACAAAATGGCAGAAAGGGATATAGTCTTGCGAAACGCCAGAATTGGGAATTTGATCATAGATTCAGTGTATCCTTTTGAAAGGACAATTAATGATGTTTTTGAAATAGGGGATATACTGATGCTCCATTTAGGAAATGATGCATATAAAGTGATAAAGTGCTATGAGGCTCTGATTACAGGAATTCTTCTCTCTAGATGTGAATCAATCATTTTAGATAAAGAAGAATTCCTTAGAGAGACTATAAGTGATTTGCTCGATGATAATCCAGAGTATGAATGGTCGATTTCAGGATGGTTATCAATAGCAAACAGAATGGCAACAGATCATCATCTATCTCAATTGTATGGATTGTATCGATTATGGGGACATCCGGTTGTAAAATCTACAGATGGATTGCAGAAAGTCAACAGGAATGGAAAGGCAAATAAATCTATCAACAAGTTGATAGCAAACATGGCAGGGATATCTTTTAAAGAGCAACTCTATTCGGGGTATAAGAAGAAGTGGGGGAGATATCCCGCTTTAAAGTTGCTATTGGATAACAGAACTGTTGAGGAATTATATGAAGAATCTTATCTCATCAAATGTCTTGTAGATAATCGATCTTTTGACACAAGAAAGGATGTATACATCCCGTCAGATTGGGATTTAGTTATCAGTCAGAAAGCACTGAGTCTACCTGAGACTTTTAATTTGACCATGGTAGTAGATGACAAGGCTATATCTCCTACCAAAGATTACCTCATATCGGTAGCATTGGGTCAAAACCGATTAATGAATCCTTTAGAGAGAAGAGGTGTGTTGAAGTGGATGAACGAGGATTATCATGATTGTCAGGCCTTCTTACAAAAGATCAATGATAACAGTCTGGACTCTAATGACTGTGTGATAGGGCTTTATCCTAAGGAACGAGAATTGAATTCTATACCCAGGATGTTTGCTCTCATGTCTGCTAAGATGAGGAACTATGTTGTTGTGACTGAGCATATGATTGCTGATGATATACTCCCTTTTTTCCCTCAGATAACGATGATGGATGATTTATTGAGTTTGACAAAGAAGATACACGGGGCAACACGCTGCCAGCAAAATAAAGTGAATGCATCAGGGCTTTTTTCAAAGAACAAGTATCTGATCCAAGTTGACATTTGTTTGAACATGGACTTTGAGAAATGGAACTTAAATATGCGAAAAGAATCCACCTATTCAGTTTTCCTAGAGATGGGGAGACTATATGGGATGGATGAGTTGTTCAACAGGACATATGACATTTTTCATGAGAGTTTTGTATATGTTAGCGACGAGAATGCAAAGTTGGAGATTGGATTGGATGACAACGGGGTCCCTCACTTGAGGCCAGACAACGTTCACTCATACACTGGACATATCGGAGGATTTGAAGGCTTGAGACAGAAGGGGTGGACAGTTTTTACGGTAGCGGTGATACAAATGGTTTTGAAAGATTTTCCGGTGGCTTACAAATTGATGGGGCAGGGAGATAATCAAGTTCTCATGTTGACTTTAAAAACCAACTCAGTTGACCAATGCGGAAATATAACTGATGATGGAATATTGGAACTAAGAGGACTATTGAAACTGGTGATTAATCGATTGGAATCAGTGTTTTTGGAACTTGGTTTGCCATTAAAGACACTTGAGTCTTGGAGGTCAGAAGAATTTTTCTTATACGGAAAATTCCCTGTGAAGAAAGGTATTCCTCTGAGTATGTCATTGAAGAAGCTAAGCAGGTCATTTCCCTTCTCAAATGACGATAGTATGACGATCGACAATGTTATGGGGTCTGTCTTTACAAATGCACAGTCTGCATCCATGTCAGACGTAACCCACTTATTGGCGTATTATTCGGGAATATTTGAGGTCATAAATGGTGCCATGTTAGTTCTAAACTGGCATCCTTTGATCGGACAAGGTTTTTTTGGTTATTTAAAAGAAGGTTTCAACTGGTTCACTTATGAAAATGTTTCATCTGATGACAATAAACGGAGCAAGAAAATATCAATAAGCATACAGGGCACAATGGAGTATTATCTCTTTATTGAATTATTATCTCTAATGCCTAAGTCTCTCGGAGTTTCAAATGGGATAACAGAATATGAATTCTTAATGAGAGGATTTCCTGATAATCAGAGCAGAGACTTGACTTACTTGTGCGAGATCATAAGTAGCAATGTTGAATCAGACAACCCAAAGGAGAGGCAAGTAATATCTGGGCTGATCAATTTTGTCAGATTCAATATTTCCAATAGCTCTAATCTTGATTTCTTGGTTGAAGATCCCTGTGCTTTAAACTTACTTCAGCCCAAAACACCAATGACCATATTAAGGAAGAAGGTCAAAGACACCTTGACTAAGACAGCCAATTTTAAAAATGAGAATTTCATGGGGTTGTTTAAGCTGTCGATAGATGAGTCCAGAAGATCATTATTAAACAAACTTGCTGAAGGGGATGTATTATTTCCTAGGGTTTTACATGATTGTTATGCAGCTTCCTTGTTTGGGTTTGTTGATGGGATAGTTTCGAAAGTGGATAAGACCGTAACTGTTCAAAGGATCTGTTTGGAGACATCAGATGATGATATAATTAAGGGGCTCTGTCTTGCTGAGAAAAATTATATCAAATATCTTTTCTGGAGATGTGAATTTTATAGGGCAGCTAAGTATGAGAACGAGCCTAAGATTTCCTGTCCCACAAATTATAGCAGATGGTTGAGAAACGCTGGATGGAAGAAGGAGGTCCAAGGAGTAACTGTTCCTTATCCTAGTCACACTTTGATGTACAGAGGTAATGATTGTTGCATATCTTGTGATGGAAACGACATCATTACCTGCCATGTATCGGATTTTCTACCAGAGACCACAGAGAGGCTGGTATCATCACTTGGTCAGTCTCCCCCGTATCTCGGAAGTTATACAAAAGAGAAAGTGAAGACATACGATAGAGTGGCACTGTACAGCTCTGAGCCGTTGTTAAGAAGAATTGTGCGTATGCTGAGGATCATAGGATGGGGTAATTTAGAGGTATCGAATTTGCACAATTATCTCAGAAATTTACTCAGGTCGGTATGCGATGTAGATGACTCAATATTTCTCTTAAATAAAGAAGATATTGGAGGGTCTCTGGAGCACAGGTATCGAGATAGTGCTTTGAAGCATGGGGCCTTAGCAAGCAACATGTATGGGCTAGGGACATGGATACACATGAGCACGGATAAGTTTGGACAATACACTAAGGGGAGCAAAAATGTCACTTTGCATTTTCAAGCTATATTATGTTGGGTGCAATCAAGAATGTATGAGATCCTCCTGGACTCATCTTTCTCGAATGGAATCGAGTTCAAGGAGTTCCACTTTCATCTCAGTTGTAGTGAATGTATAAAAAGTGTTGAGTATGATGTTCCAGACATCTCTTCAGTCCCTAAAGCTTTGATACCGAAGTTGAAAGATAACCCTTATTGTTATGTTAAGAATGTGATGCTAACTGAAAAAGATAGGTCCGTATATGCAATAAAGGATATCTTTCAGATAGACTCTAATATTGGAATACATAATCTCGATAGCAAAAGTATCAAGTACTTATTTCATGAGTTTTGGGCTGCATCGATATCCAGAGATATCTTTTCTACCTCAGAGACAGATAACACGTCAATAGGAACAGGGGTCCTCGAGATAAATAAATACCCTAGAATTGCATTCTACAGAATCGGAGCTGAACTTTTATATGATTTGATAAGTGACATATCTATCCTATCAGTCATGCGAATGGAAGCGGAGAAGGACACTTACAAAGAATCCGCGATCTCGTTAGACCATTGTGTTAGAGTGTTACTCAATACTTTGAATGAATGTGACAATGAGGGGTTTATCGGGTTGAGTATCCTTTTTTCATGGGAAAATCAAATAAAAGAAATCATGAATTTTACTTCAGTTCTCTTGCCGGGCAGTGTTTCTATGTCTGTAGGTGATTGCTTATTGGCTGCAAAAAGATCATTAATTAATTATGGGGAAAATAAGACAAATTGGTTCAACCACAGATGCAGATATATCAACTTGAATGAGATGACCCCTGTGGATACATCTATACTCTTGAGGTACGCTGCAGAATTTGATTTCGAATCAGTAGAATCCTGTTCTGAGTGTCGAAGGAGTATCTTGCTTAATATATCAAATGAAAAGCTGTTGTCTTTATCAGGAACAGAGAAATGTGAGTATGGTCATATTTGGTTTCATAAGGAGTATACCACTAAATATGTGACTGTGATTTGCATTCCAGAAGATGCATTGTCTAAACAACAATCATTCAGTTATCTTATAAAGACAAAGGTCGTCAAGCAGTCAATCAGAAATAAAGAGTTAAGAAACCATTGTAATACTATGAGACGGTGTTTTTCTCTGAAACCTGTGTTCTCATCAAATGATATTAGATACACTAATGATCCCTCGATAATAGAAGGCAAGATCTTGTTCCAGGGGAATGTGGATCCCATGAGATTGTGCTATAGATGCATACCTACTGAATTGTCATCGGGATATAGATTATTGGACATATTGGTCGGTTTACGTATTCCCGTCATGGAAAGATCTTGCAGAGTCCTTAATTTAGGGGATGGATTTGGCGGAACAGGGTATTTGCTGGAGAAGCTGATAAGATGTCATGTGATAAATGCAACGTTAGTTGATTGCACTAGTGCTTTTCCACAGACATTTCCTAACTCTAGACCATCTAGTCAATACCTGAGTAATAATTTGTCTCGGTTCGATAATTCCTTAAGCAAGATAATGGTTAATAATATATTTTCTGAAGCGGTTGTCTTAAGGTACAGGCAGGTATGCGAGTTAAGTGGTACAAAATTGGGCATTTGTGATATTGAACTGGAACACAATGAGACTTCAAGCTTCAACAATGTTGGCCGATTCCCATATTCAAGACTGATAAAGCAGTTAAGAAAGATCAATTTAGACATATTCTTAGTTAAGATCCGGGTCAAAAGCAATGTTGAACTTTATCATGTCTTAGAAATTGCAAATTATAACTATCATCATTTTGAGGTCTATATCACCCCGACTTGCAACAACATGAAGGGAGAATTCTTCTTAAGGTTGGAGGGAGCAAAAGAAGAGTTGCCTATAGAAAGTCGGATAATGAATTTCTCCACTAGAAATAAGATATTAAGGCGTTTTATCAATTATACTGGAGGGAAGTCTTTCTCTAATGTTGATAGTGAATACTATTTTGCTCAGAATCTTATCATGATAGACCCGACATTGCAACATAGAATGCTATCTTATGTAAAAGATTGGATAAAGGAACCGAGATTGAACATAGATATGAGCATGAAAAGTTTCACATCTTTGTTCTTATCAATTGCCGCTGCAAGAAATCCCCGATTGTTTGTCCATCACACAGATAAGAAAGACAAGTATGAGTATAATGATGATCTTTTGAGTATTGGATGGAGGATCTTTACTCTTGGGCTTGCAAAAATACAATCTGAGTCAACATGTTGTTCTGCACTTGATAACATTGAGAGTTACCTGCTAATCAGACCTGCTGTGGTTTCAACTGTAAATAAGGTAAAGGTGATAAGAAAATACACTTATATGATAGT
