# Supplementary material for: Identification and characterization of a novel Cytorhabdovirus associated with goji berry (Lycium barbarum L.) crinkle disease
Source: Front Microbiol. 2024 Jan 4;14:1294616. doi: 10.3389/fmicb.2023.1294616 (PMC10794335; doi:10.3389/fmicb.2023.1294616)
Supplement: Supplementary file 4 [file Table_4.docx]

Table S4. The pairwise comparison of goji cytorhabdovirus A isolates NX (GCVA-NX) and BJ (GCVA-BJ), and yerba mate virus A (YmVA)

| Virus |  | Genome |  | N | P | P4 | M | G | L |
| --- | --- | --- | --- | --- | --- | --- | --- | --- | --- |
|  |  | nt identity |  | nt identity /aa identity | | |  | | |
| GCVA-NX/ GCVA-BJ |  | 98.93% |  | 98.8%/98.32% | 98.64%/98.06% | 98.75%/97.91% | 99.07%/100% | 98.88%/96.63% | 99.18%/99.35% |
| GCVA-NX/ YmVA |  | 43.76% |  | 44.67%/31.38% | 39.31%/22.08% | 45.79%/30.08% | 37.01%/12.87% | 40.56%/17.54% | 50.24%/39.89% |
| GCVA-BJ/ YmVA |  | 45.14% |  | 46.63%/35.82% | 42.64%/22.34% | 45.65%/30.08% | 36.84%/12.87% | 40.00%/17.54% | 49.85%/40.86% |

goji cytorhabdovirus A isolate NX (GCVA-NX, OR489165), goji cytorhabdovirus A isolate BJ (GCVA-BJ, OR489165), yerba mate virus A (YmVA, NC_076472)
