# Supplementary figures and images for: Identification and characterization of a novel Cytorhabdovirus associated with goji berry (Lycium barbarum L.) crinkle disease
Source: Front Microbiol. 2024 Jan 4;14:1294616. doi: 10.3389/fmicb.2023.1294616 (PMC10794335; doi:10.3389/fmicb.2023.1294616)

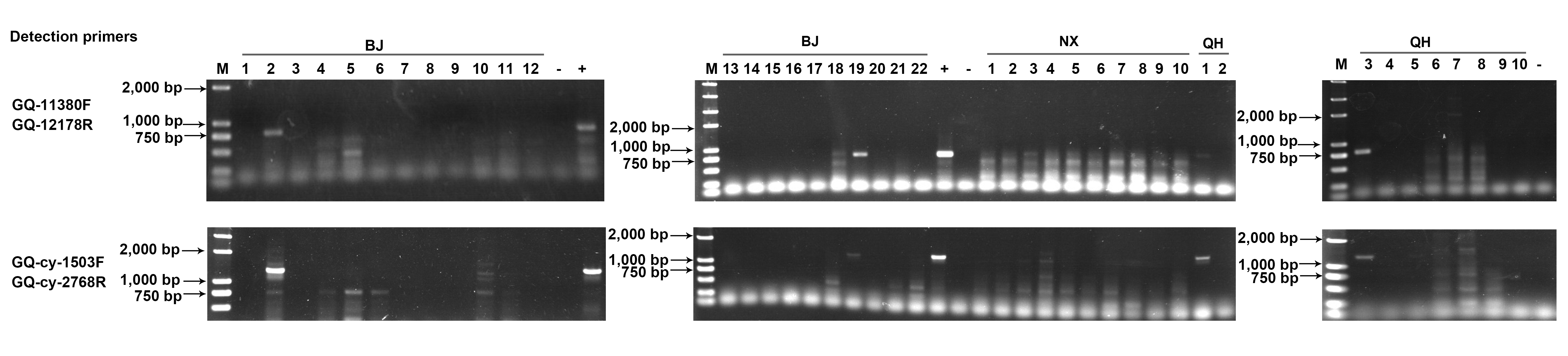

Supplement: Supplementary file 5 [file Image_1.jpeg]
